# Long-range gap junctional signaling controls oncogene-mediated tumorigenesis in *Xenopus laevis* embryos

**DOI:** 10.3389/fphys.2014.00519

**Published:** 2015-01-19

**Authors:** Brook T. Chernet, Chris Fields, Michael Levin

**Affiliations:** ^1^Department of Biology, Tufts Center for Regenerative and Developmental Biology, Tufts UniversityMedford, MA, USA; ^2^Independent ResearcherSonoma, CA, USA

**Keywords:** cancer, connexin, ion channel, membrane voltage, bioelectricity, long-range signaling, left-right asymmetry

## Abstract

In addition to the immediate microenvironment, long-range signaling may be an important component of cancer. Molecular-genetic analyses have implicated gap junctions—key mediators of cell-cell communication—in carcinogenesis. We recently showed that the resting voltage potential of distant cell groups is a key determinant of metastatic transformation and tumor induction. Here, we show in the *Xenopus laevis* model that gap junctional communication (GJC) is a modulator of the long-range bioelectric signaling that regulates tumor formation. Genetic disruption of GJC taking place within tumors, within remote host tissues, or between the host and tumors significantly lowers the incidence of tumors induced by KRAS mutations. The most pronounced suppression of tumor incidence was observed upon GJC disruption taking place farther away from oncogene-expressing cells, revealing a role for GJC in distant cells in the control of tumor growth. In contrast, enhanced GJC communication through the overexpression of wild-type connexin Cx26 increased tumor incidence. Our data confirm a role for GJC in tumorigenesis, and reveal that this effect is non-local. Based on these results and on published data on movement of ions through GJs, we present a quantitative model linking the GJC coupling and bioelectrical state of cells to the ability of oncogenes to initiate tumorigenesis. When integrated with data on endogenous bioelectric signaling during left-right patterning, the model predicts differential tumor incidence outcomes depending on the spatial configurations of gap junction paths relative to tumor location and major anatomical body axes. Testing these predictions, we found that the strongest influence of GJ modulation on tumor suppression by hyperpolarization occurred along the embryonic left-right axis. Together, these data reveal new, long-range aspects of cancer control by the host's physiological parameters.

## Introduction

Consistent pattern formation during embryogenesis and organ regeneration requires the coordination of cell activities and information across large distances *in vivo*. Alongside gradients of extracellular secreted molecules, functions an important system of direct cell to cell communication. Gap junctions are intercellular channels that allow small molecule-mediated signals to pass directly from the cytoplasm of one cell to its neighbors (Goodenough et al., 1996; Bruzzone et al., 1996a,b). Connexins, subunits that make up gap junctions (GJs), control the transfer of a wide range of cellular molecules based on their charge, size, and shape (Loewenstein, 1979, 1981; Goldberg et al., 2004), serving as true electrical synapses among non-neural cells (Bennett, 1997; Pereda et al., 2013; Rash et al., 2013). Due to their rich combinatorial properties and gating regulation, gap junction function can implement complex circuits with properties such as memory (Palacios-Prado and Bukauskas, 2009; Pereda et al., 2013). Many biological processes have evolved to take advantage of these versatile elements, using GJs to regulate differentiation, proliferation, and apoptosis (Krysko et al., 2005; Wong et al., 2008), as well as morphogenetic signaling in many contexts, including development of the brain, limb, bone, tooth, heart, and the left-right axis (Levin, 2007; Wong et al., 2008). Not surprisingly, loss of morphogenetic control due to defective gap junctional communication (GJC) has been implicated in numerous disorders, including hearing impairment (Rabionet et al., 2000), neural tube defects (Ewart et al., 1997), parasitic infection (Vega et al., 2013), cardiovascular diseases (Jongsma and Wilders, 2000), and other genetic syndromes (Zoidl and Dermietzel, 2010). Interestingly, while GJC is most often thought of as mediating local interaction, it can also provide long-range morphogenetic coordination on the scale of the whole organism, for example during the establishment of polarity in regenerating planaria (Oviedo et al., 2010) and left-right pattering of *Xenopus* and chick (Levin and Mercola, 1998, 1999).

A major area in which GJC has been implicated is tumorigenesis (Yamasaki et al., 1995, 1999; Ruch and Trosko, 2001; Trosko, 2005; King and Bertram, 2005; Mesnil et al., 2005). A role for GJs, as mediators of cell-cell signaling and information exchange, is consistent with a view of cancer as a developmental disorder—a derangement of the interaction of cells with the normally tight field of patterning controls of the body (Tsonis, 1987; Pierce and Speers, 1988; Clark, 1995; Dean, 1998; Rubin, 2006; Bissell and Hines, 2011; Marongiu et al., 2012). Alongside cell-autonomous mutations in so-called cancer stem cells, tumorigenesis and progression are controlled by biophysical properties of the surrounding microenvironment (Chernet and Levin, 2013a) and even by neural inputs (Scharrer, 1953; Pawlowski and Weddell, 1967; Magnon et al., 2013). The molecular nature of the processes by which surrounding cells regulate transformation and metastasis is an important area of research today.

By regulating the spread of morphogenetic signals, GJs are an ideal candidate for keeping individual cell activities coordinated toward the anatomical needs of the host, or conversely, allowing confounding signals that may induce tumorigenesis (Levin, 2011, 2012b). Disruption in the function of GJs is implicated in a number of cancers (Yamasaki et al., 1995; Duflot-Dancer et al., 1997; Yamasaki et al., 1999; Ruch and Trosko, 2001; Mesnil et al., 2005; Sirnes et al., 2012). For example, Cx26-deficient mice exhibit a 25-fold increased incidence of spontaneous liver tumors (Temme et al., 1997). Moreover, tumor incidence is higher, and clinical prognosis is worse, when cells are gap-junctionally isolated by pharmacological agents or genetic mutation (Loewenstein and Kanno, 1966; Loewenstein, 1979; Rose et al., 1993; Mesnil et al., 2005).

Most clinically-relevant tumor cells are known to exhibit down-regulation in connexin expression, leading to the disruption of effective cell:cell communication (Soroceanu et al., 2001; Gee et al., 2003; Mesnil et al., 2005; Talbot et al., 2013). However, in some some studies, enhanced GJC was suggested as a tumor promoting factor (Saito-Katsuragi et al., 2007; Naoi et al., 2007; Elzarrad et al., 2008; Haass et al., 2010). Breast cancer and melanoma cells take advantage of these connexins to enhance their metastatic potential in Stoletov et al. (2013). Together, the data suggest that it is imperative to understand the signaling mediated by GJs and the information passed among normal cells that could promote (Rose and Wallingford, 1948; Lewalle et al., 1998; Zhang et al., 2003; Donahue et al., 2003), or normalize (Hendrix et al., 2007), cancer *in vivo*. How might GJC regulate tumorigenesis in the context of tissue and organ patterning?

One carrier of the morphogenetic cues that go awry during tumorigenesis may be current—the movement of charged ions. Gradients of resting potentials established by ion channels and pumps in the cell membrane are now known to be instructive patterning cues that regulate cell behavior during pattern formation (Levin, 2012a; Tseng and Levin, 2013; Levin, 2013, 2014), and gap junctions sculpt the distribution of iso-potential cell fields by allowing specific cells to equalize their trans-membrane voltages (V_mem_) in response to various physiological signals. Bioelectric gradients have already been implicated in the control of metastasis (Morokuma et al., 2008; Blackiston et al., 2011) and oncogene-mediated tumorigenesis (Lobikin et al., 2012; Chernet and Levin, 2013a,b, 2014), while specific ion channels are becoming increasingly recognized as oncogenes and important drug targets (Diss et al., 2005; Fraser et al., 2005; Arcangeli et al., 2009; House et al., 2010; Yildirim et al., 2012; Arcangeli et al., 2012; Yang and Brackenbury, 2013; Than et al., 2014; Pardo and Stuhmer, 2014). Interestingly, the resting potentials of *distant* cells are critical for oncogene-dependent tumorigenesis: modulation of ion channels in locations quite distant to oncogene expressing cells in *Xenopus* tadpoles significantly reduces the incidence of tumors. This effect is mediated by a butyrate-based mechanism that regulates oncogene-mediated tumorigenesis via histone deacetylase activity (Chernet and Levin, 2013a,b, 2014), but the spatial dynamics of butyrate signaling in this context remain to be elucidated. The known role for ion flows and resting potentials in cancer suggests the possibility that gap junctions participate in bioelectric regulation during carcinogenesis and/or neoplastic progression.

To investigate the complex interplay of physiological and genetic signals in tumorigenesis, we pursued a combination of modeling and experiment to probe the spatial relationships between tumorigenesis, voltage properties, and GJ paths *in vivo*. We recently showed that ion channel-mediated changes in V_mem_ can modulate the tumorigenicity of human oncogenes misexpressed in the *Xenopus laevis* embryo (Chernet and Levin, 2013b, 2014). Here, we use this assay to investigate the interplay between bioelectric controls and gap-junctional connectivity *in vivo*. Unexpectedly, we found that disruption of cell:cell communication via H7—a chimeric connexin construct that is known to inhibit GJC in *Xenopus* (Paul et al., 1995; Levin and Mercola, 1998)—is able to suppress tumor formation. Remarkably, the suppression effect was observed regardless of where GJC disruption was occurring (host-wide, within tumors only, or away from tumors). Indeed, the most pronounced suppression was recorded for GJC disruption taking place non-locally to oncogene-expressing cells, revealing a role for distant cell:cell communication in tumorigenesis. Conversely, enhanced GJC within tumors or their microenvironment, achieved via the mis-expression of a constitutively permeable junction-forming connexin Cx26 (Levin and Mercola, 1998), increased tumor incidence. Together, these data suggest that GJC is a mediator of both local (within tumors) and long-range (within the microenvironment and the host) signaling and that specific patterns of physiological isolation may be necessary for tumor suppression. Here we also formulate a mechanistic, quantitative model consistent with these data and with prior work on endogenous left-right asymmetric voltage gradients, and test key predictions of this model. Based on these data, we suggest that exploiting the bioelectrical signaling that occurs through electrical synapses among somatic cells represents an important target for cancer therapy that is distinct from the targeting of individual channels.

## Materials and methods

### Animal husbandry

*X. laevis* eggs were fertilized *in vitro*, and embryos were cultured according to standard protocols (Sive et al., 2000), in 0.1X Modified Marc's Ringers (MMR; pH 7.8) with 0.1% Gentamicin. *Xenopus* embryos were housed at 14–18°C and staged according to Nieuwkoop and Faber (1967). All experimental procedures involving the use of animals for experimental purposes were approved by the Institutional Animal Care and Use Committees (IACUC) and Tufts University Department of Lab Animal Medicine (DLAM) under the protocol number M2014-79.

### Microinjection

Fertilized *Xenopus* embryos were transferred into mesh-bottomed dishes with 3% Ficoll and injected with capped, synthetic mRNAs (made using the Ambion Message Machine kit) dissolved in water at the stages indicated. The doses per cell were *KRAS^G12D^* (Le et al., 2007), 40 pg; H7 (Paul et al., 1995), 70 pg; Cx26 (Levin and Mercola, 1998), 500 pg; and β-gal (lineage tracer), 400 pg. Two hours after injection, embryos were transferred into 0.75X MMR for 45 min before they were washed and cultured in 0.1X MMR until desired stage was reached. *KRAS^G12D^*-injected embryos were raised to stage ~35, and scored for the presence of tumors using bright field microscopy as described in Chernet and Levin (2013b).

### Testing GJC

**Figure 1:**
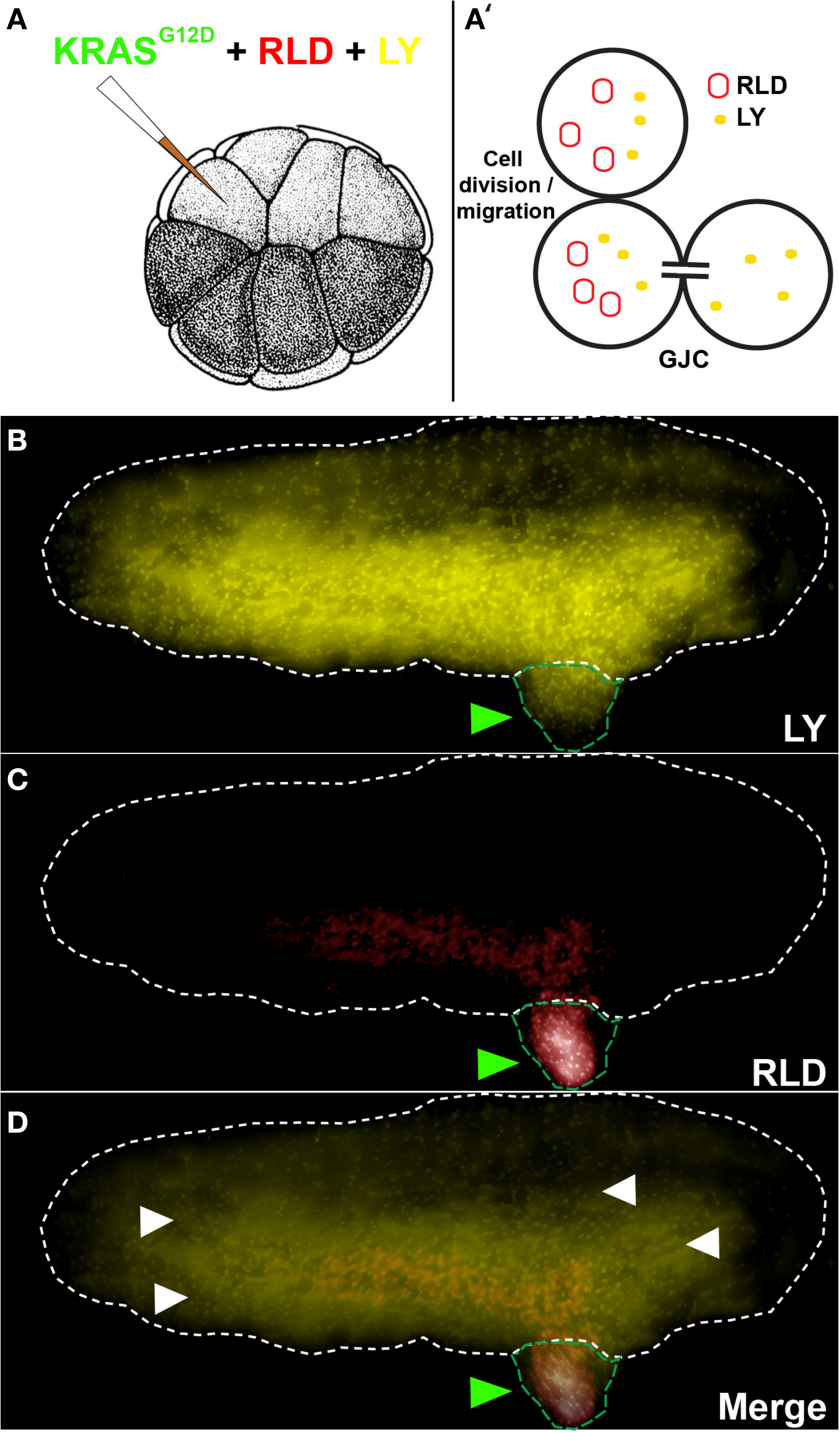
***KRAS^G12D^* injection results in the formation of induced tumor like structures that are gap junctionally connected to the host. (A)** The schematic shows injection of *KRAS^G12D^* + RLD (Rhodamine-Lysinated Dextran, M. W. 10 kDa) + LY (M.W. 0.522 kDa) into one cell of a 16-cell stage embryos. The RLD labels injected cells (and their descendants), while LY is small enough to traverse gap junctions. **(B)** At a stage 34 embryo (white dotted outline), LY signal is present not only in tissue derived from the injected cell (tumor; green arrowheads) but also widely throughout the rest of the host. **(C)** RLD shows cells injected with oncogene, some of which are in the localized tumors and others that have migrated (metastasized) out into the host. **(D)** Overlay of the two signals reveals areas (white arrowheads) that exhibit LY signal but not RLD signal, which demarcate cells that are in active GJ communication with the injected (tumor) cells (*N* = 5 of 5). Schematic in panel A (and in subsequent figures) is used with permission from Nieuwkoop and Faber (1967).

GJC was assessed between tumors and the host using a 1:1 mixture of Rhodamine-Lysinated Dextran (RLD, 10 kDa, Life Technologies) and Lucifer Yellow (LY, 0.522 kDa, Life Technologies) (Figure 1). 16 cell embryos injected with *KRAS^G12D^*, RLD, and LY were allowed to grow to stage ~35. Cells exhibiting LY signal (which passes through GJs) in the absence of RLD (which does not pass through GJs) signal reveal an open gap-junctional communication (cells with both LY and RLD signal indicate regions that have acquired the two molecules through cell division and/or migration). RLD and LY were detected in live embryos using TRITC and Lucifer Yellow filtersets, respectively, on an Olympus BX61 spinning-disk confocal microscope with Hamamatsu ORCA digital CCD camera.

### Statistical analysis

Data were expressed as the mean unless otherwise noted. The differences between treatment groups were analyzed using Student's *t*-test and *X*^2^ test, and the null hypothesis was rejected at the 0.05 level.

### Predictive modeling

A quantitative model of embryonic growth and response to microinjection treatments was developed in two stages that model different processes (**Figures 4, 5**). The first-stage “left-right synchronization” model was implemented using JavaScript and the HTML5 “canvas” function. Details of this model are described below (Section *A two-stage quantitative model describes the dynamics between V*_mem_, *GJC, and tumor formation*); the model is interactive, and can be manipulated and the source code can be examined at http://chrisfieldsresearch.com/convergence-demo.htm (also seen in Supplement 1). The second-stage “left-right communication” model comprises the implications of a set of assumptions that specify a control network as described below (Section *A two-stage quantitative model accurately predicts the dynamics between V*_mem_, *GJC, and tumor formation*). Numerical predictions were calculated manually from these assumptions.

## Results

### KRAS*^G12D^*-induced tumors are junctionally coupled to normal host cells

To study the role of GJC in oncogene-mediated tumorigenesis, we made use of a simple assay that utilizes expression of an exogenous human oncogene in *X. laevis* embryos (Figure 1A). This model system is ideal for probing connexin-based GJC (Swenson et al., 1989; Barrio et al., 1997; Cao et al., 1998; Lee et al., 2009) and has been used to study GJC-mediated morphogenesis and large-scale patterning (Warner, 1992; Levin and Mercola, 1998; Levin, 2002). The oncogene *KRAS^G12D^* (Le et al., 2007) was used to induce *Xenopus* tumors (Figures 1B–D, green arrowhead). These exhibit the same major hallmarks of tumors as do their mammalian counterparts: increased mitotic activity, induced vasculogenesis, disorganization of normal cellular architecture, increased hypoxia, acidic microenvironment, and ability to illicit innate immune response (Chernet and Levin, 2013b, 2014). We began by testing GJC between induced tumors and the host, which revealed considerable spreading of the small GJC-permeant tracer Lucifer Yellow (Figures 1B,D; yellow signals, white arrowheads) away from descendants of one of the 16 progeny cells that were injected with oncogene mRNA and a large, GJC-impermeant Rhodamine Dextran (5 out of 5 tested; Figures 1C,D; red signal). Having observed active GJC between the tumor and surrounding host tissue, we began functional studies by investigating how the growth of these oncogene-induced tumors is affected by spatially targeted disruption of GJC. All of the reagents used below were titered to levels low enough that the normal background processes of embryogenesis and growth proceeded normally.

### Disruption of GJC reduces tumor incidence

**Figure 2:**
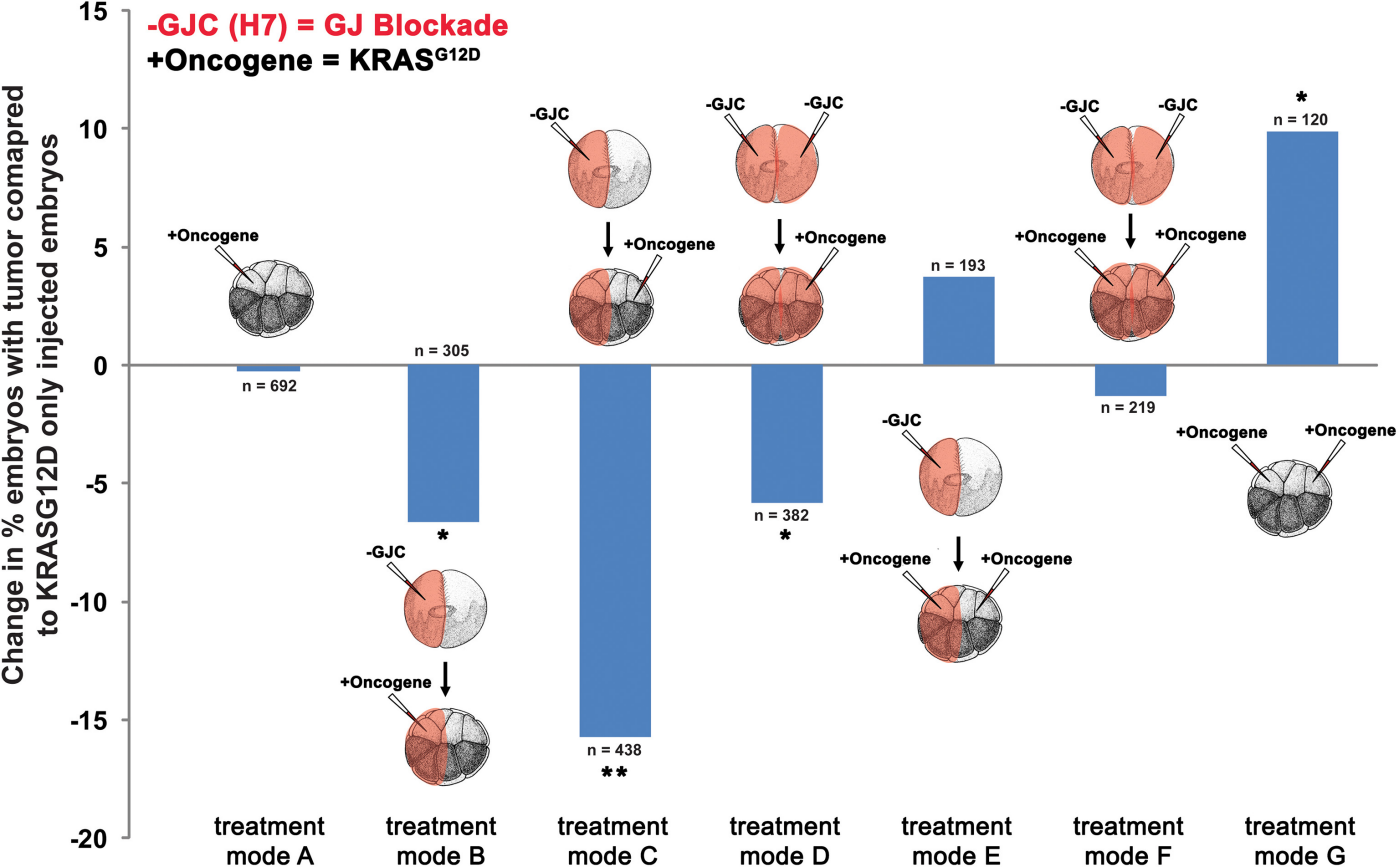
**Selective disruption of gap junctional communication (GJC) reduced tumor incidence**. To determine the effect of GJC disruption on tumorigenesis, H7 and *KRAS^G12D^* injections—aimed at disrupting GJC host-wide, within tumors, and away from tumors—were performed. Compared to *KRAS^G12D^*-only injected embryos (treatment mode A), treatment modes B and D showed significant decrease in % embryos with tumor by 6.6 and 5.8%, respectively, implying that H7 contralateral to the oncogene has no effect if there is H7 ipselateral to the oncogene. Contralateral H7 only (treatment mode C) significantly reduces the % embryos with tumor by 15.8%. Doubling the level of oncogene injected along with host-wide introduction of H7 (treatment mode F) does not affect tumor incidence when compared to oncogene-only (one side) injected embryos (treatment mode A), but is able to counter the 9.9% increase in tumor incidence resulting from excess oncogene introduced (treatment mode G) down to the tumor incidence level of one-side oncogene injection. Similarly, tumor incidence from the excess oncogene introduction can be reduced by H7 introduction to either side of the embryo (treatment mode E). ^*^P < 0.05, ^**^P < 0.001; *t*-test.

To determine the effect of GJC disruption on tumorigenesis *in vivo*, mRNAs of dominant-negative connexin H7 (Paul et al., 1995; Levin and Mercola, 1998) and *KRAS^G12D^* were injected in *X. laevis* embryos. The oncogene and GJC disrupting constructs were introduced in different spatial arrangements to investigate what happens when GJC is disrupted host-wide, within tumors, and away from tumors. Taking advantage of the embryonic fate-map (Dale and Slack, 1987; Moody and Kline, 1990), we were able to control which cells received oncogene mRNA and which cells were inhibited with respect to GJC. To achieve suppression of GJC within the tumor (“local” mode), H7 mRNA was injected into 1 cell of a 2-cell embryo and the *KRAS^G12D^* mRNA was introduced into one of the progeny of that cell (at the 16-cell stage). For remote (“long range” mode) GJC suppression in tissue outside the tumor, H7 mRNA was injected into 1 cell of a 2-cell embryo, which was raised to the 16-cell stage and then injected with *KRAS^G12D^* mRNA into one of the cells on the opposite side of the embryo from the cells bearing the H7. For host-wide GJC disruption, both of the 2 cells of the 2-cell embryo were injected with H7, and *KRAS^G12D^* mRNA was then injected in 1 of the 16 progeny cells. Figure 2 shows the various combinations of which sides of the embryo received the GJ blocker (H7) and which received the oncogene (KRAS*^G12D^*).

Compared to tumor incidences observed in the baseline *KRAS^G12D^*-only injected embryos (Figure 2; treatment mode A), local (treatment mode B) and host-wide (treatment mode D) disruption of GJC showed significant suppression of tumor formation by 6.6 and 5.8%, respectively (*t*-test; *p* < 0.05). Interestingly, long-range disruption of GJC (treatment mode C) had the strongest suppressive effect, showing a 15.8% decrease in the number of embryos with tumor (*t*-test; *p* < 0.001). Injection of H7 host-wide with *KRAS^G12D^* injected in 2 cells on opposite sides of the 16-cell embryo (Figure 2; treatment mode F) did not affect tumor incidence when compared to *KRAS^G12D^*-only injected embryos (Figure 2; treatment mode A). However, the host-wide H7 introduction (Figure 2; treatment mode F) reduced tumor incidence resulting from oncogene expression on both sides of the embryo (compare to Figure 2; treatment mode G) by more than 11% (*t*-test; *p* < 0.05). Similarly, tumor incidence from doubling the oncogene level can be reduced by the introduction of H7 to only one side of the embryo (treatment mode E). Together, the results imply that GJC disruption suppresses *KRAS^G12D^*-induced tumors: the most pronounced effect on tumor incidence is observed when H7 is introduced at a distance from the oncogene-bearing cells, unless H7 is also expressed *within* the oncogene-expressing cells, in which case suppression is not as effective.

### Facilitated GJC promotes tumor formation

**Figure 3:**
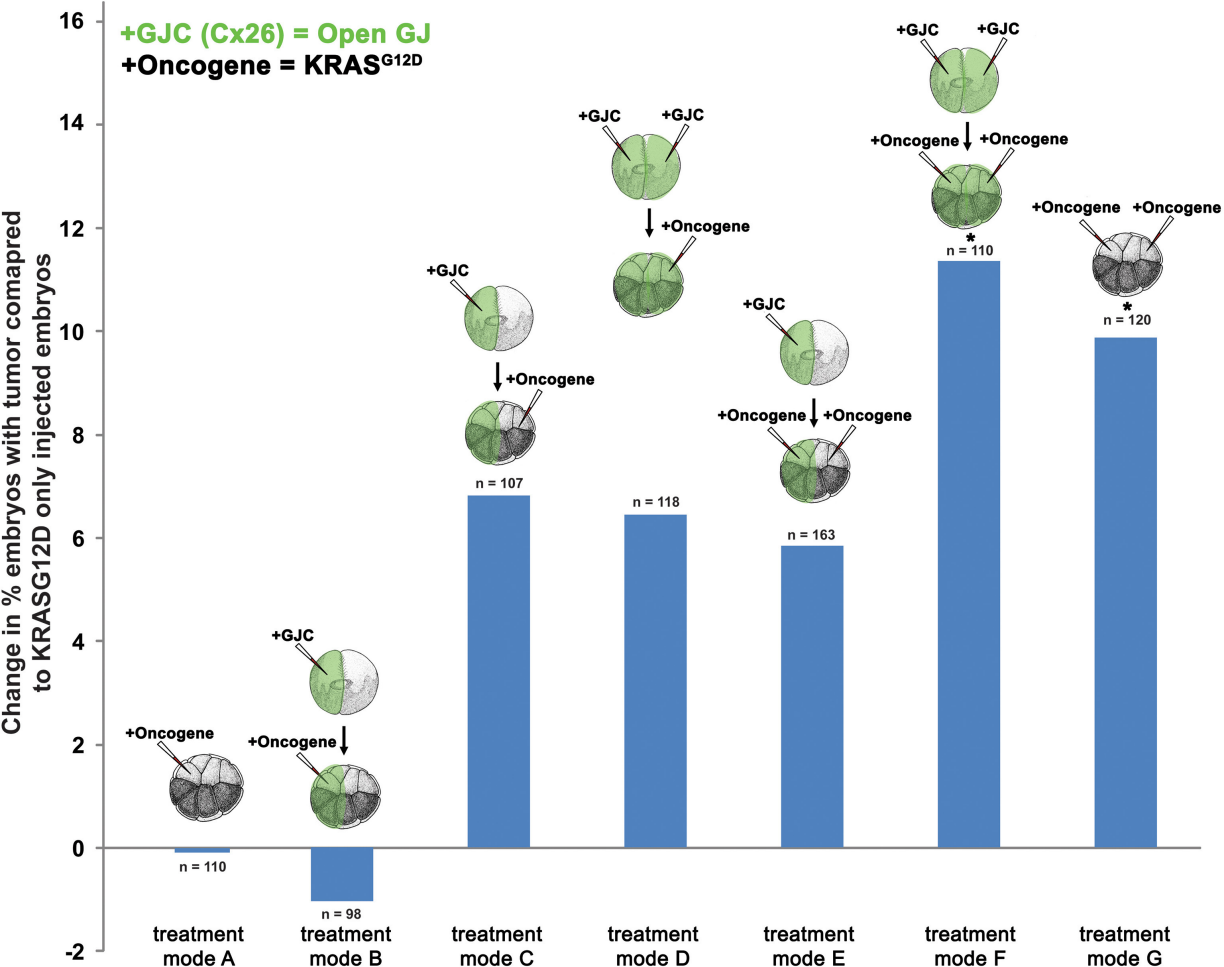
**Enhanced gap junctional communication (GJC) through connexin 26 (Cx26) overexpression increases tumor incidence**. To determine the effect of GJC disruption on tumorigenesis, Cx26 and *KRAS^G12D^* injections—aimed at enhancing GJC host-wide, within tumors, and away from tumors—were performed. Compared to *KRAS^G12D^*-only injected embryos (treatment mode A), long-range and host-wide Cx26 treatment modes (C, D, F) show an increase in the number of embryos with tumor by 6.4 to 11.4%. However, treatments involving the expression of oncogene and Cx26 on the same side (treatment modes B and E), while non-significant, lower tumor incidence when compared to treatment modes A and F, respectively. ^*^P < 0.05; *t*-test.

To further test the hypothesis that GJC plays a role in oncogene-mediated tumorigenesis, we scored tumor incidence in embryos injected with Cx26, a short connexin protein which lacks most of the intracellular regulatory region, and thus facilitates GJC by forming constitutively permeable gap junctions (Mesnil et al., 1997; Levin and Mercola, 1998). Similar to the H7 (GJ blockade) experiment, mRNA injections of Cx26 and *KRAS^G12D^* aimed at enhancing GJC host-wide, within tumors, and away from tumors were performed (Figure 3). Compared to *KRAS^G12D^*-only injected embryos (Figure 3, treatment mode A), long-range and host-wide Cx26 treatments (treatment modes C, D, F) both showed an increase in number of embryos with tumor by 6.4 to 11.4%. This is in contrast to the H7 data where modulation of long-range GJC had the most impact on tumor incidence. Interestingly, enhanced GJC within tumors (treatment modes B and E) slightly (but not significantly) decreased tumor incidences when compared to treatment modes A and F, respectively. Together, these data indicate that facilitating GJC host-wide and in the tumor microenvironment enhances the tumorigenesis process. In contrast, enhanced GJC within oncogene-expressing cells had minimal effect on tumor formation.

### A two-stage quantitative model describes the dynamics between V_mem_, GJC, and tumor formation

The tumor-incidence results were challenging to explain for the following reason. While perturbing GJC in the same side as *KRAS^G12D^* expression (Figure 2, treatment mode B) reduced tumor incidence, the effect became more pronounced when the GJC perturbation is on the opposite side of *KRAS^G12D^* injection (treatment mode C); it is puzzling why having H7 on the opposite side from *KRAS^G12D^* injection could make the *KRAS^G12D^* expressing side much less likely to form a tumor. More strikingly, why would perturbing GJC on both sides of the embryo, while only introducing *KRAS^G12D^* on one side (Figure 2, treatment mode D), have the same effect as perturbing GJC on the *KRAS^G12D^* injected side only?

**Figure 4:**
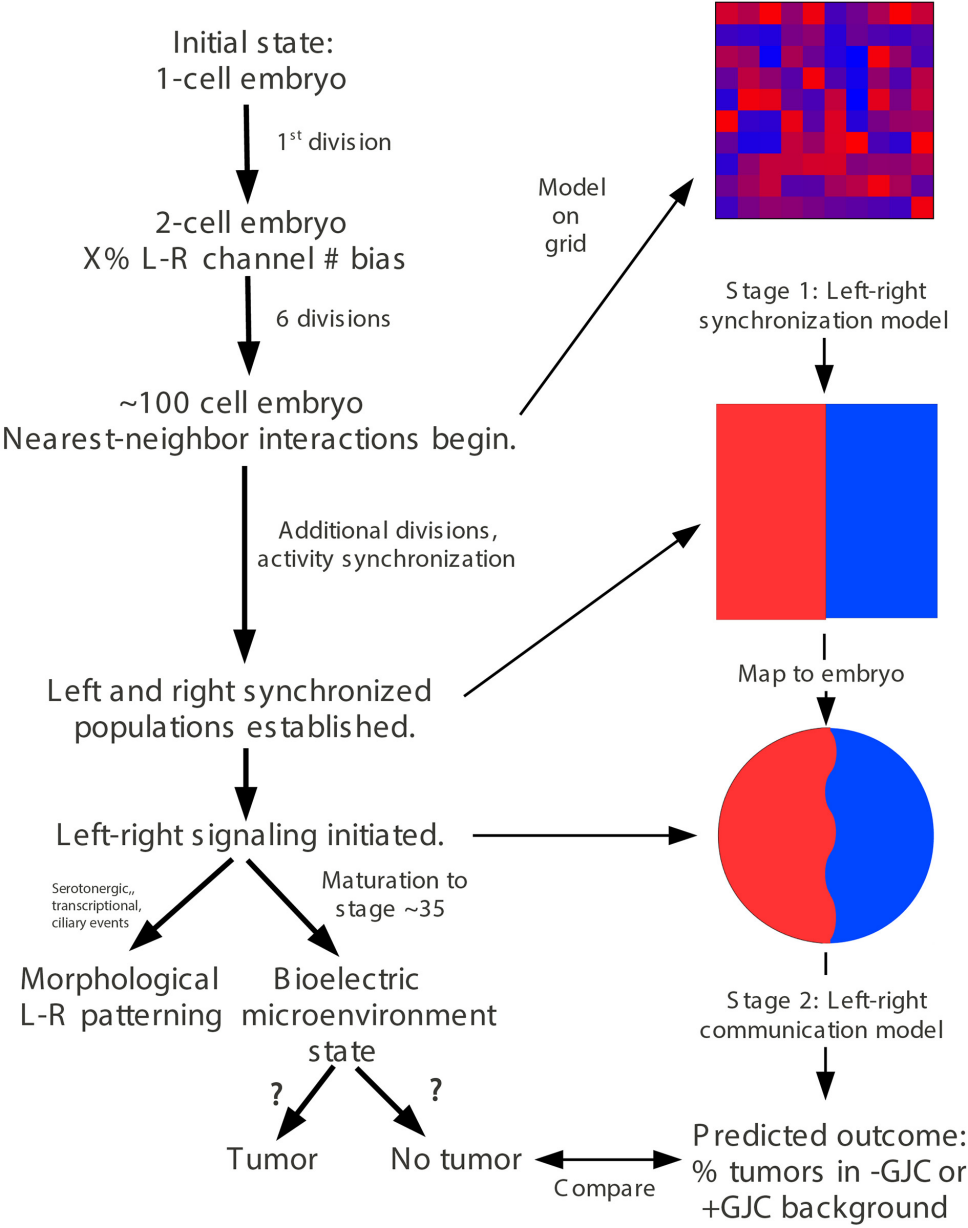
**“Left-right synchronization” model for the establishment (Stage 1) of electrical states that modify long-range gap-junction-mediated influences on tumorigenesis**. The “left-right synchronization” model shows that stable, bioelectrically-synchronized cell populations on the left and right sides of the embryo, here represented by red and blue regions, can be generated from a random initial state by nearest-neighbor interactions alone. This model is implemented on a fixed 10 × 10 grid with the left and right exterior boundaries mathematically identified to yield a cylindrical topology. This topology allows the two synchronized populations to “wrap” around the exterior border. The final model shown here was obtained by varying the left—right polarization bias parameter “X” while requiring two stable populations with no ectopic islands as output. This procedure allowed the value of the left—right polarization bias to the predicted to be 27%, consistent with observations (Levin et al., 2002; Adams et al., 2006). While the left-right border is represented at low resolution in the model and most solutions produce a straight, sharp boundary, this is not a biologically-meaningful constraint and the actual border between “left” and “right” cell populations in the embryo may be irregular as shown. The “left-right communication” model assumes that the two bioelectrically-synchronized sides exchange a long-range, oscillatory bioelectric signal; whether this signal requires an additional trigger for initiation is currently unknown. This model employs a small set of assumptions to quantitatively predict the tumor incidence expected when GJC is either suppressed by H7 or enhanced by Cx26. These predictions are then compared with experimental tumor frequencies (indicated by “?”). See Figure 5 for additional details of this model.

**Figure 5:**
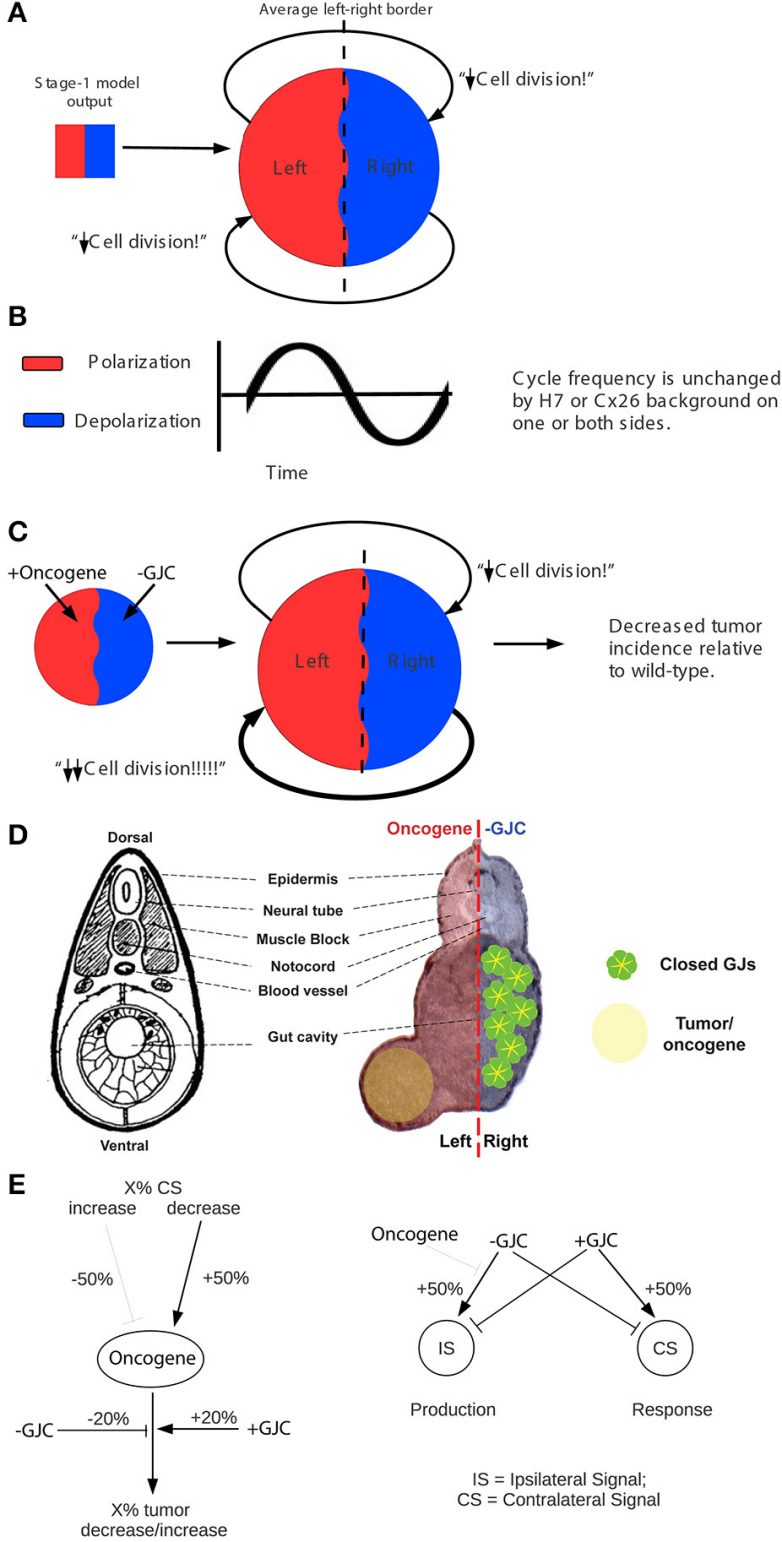
**The stage-2 “left-right communication” model for the GJC-mediated effects of V_mem_ change on incidence of tumorigenesis**. The “left-right communication” model assumes that the left and right sides of the embryo alternately signal each other to turn the rate of cell division down **(A)** using an oscillation between polarization and depolarization as the long-range bioelectric signal **(B)**. For example, *KRAS^G12D^* and H7 expressions on opposite sides of the embryo (as shown in Figure 2 treatment C, and sections through a trunk tumor in Panel **D**) result in an increase in signaling from the H7-injected side and a consequent suppression in *KRAS^G12D^*-induced tumors on the opposite side **(C,D)**. The stage-2 model can be visualized as a control network that regulates cellular response to *KRAS^G12D^* and both the production of ipsilateral signal (IS) and response to contralateral signal (CS). Here unlabeled flat-end arrows indicate a 100% suppressing effect **(E)**. In this model, gap junctions are a central component, contributing to the long-range dynamics by allowing cells to sense neighbors' V_mem_. Cellular mechanisms corresponding to these arrows are not yet fully characterized; however, the butyrate—histone deacetylase pathway previously characterized in these embryos (Chernet and Levin, 2013a,b, 2014); is a plausible candidate for transducing the global bioelectrical signal into a local cellular response to *KRAS^G12D^* transformation.

In order to mechanistically integrate the main components of our experiments (V_mem_ manipulated by ion channel injections, GJC manipulated by Cx injections, and long-range effects on tumors induced by oncogene injections), a two-stage model was developed to explain the tumor-incidence data shown in Figures 2, 3 and to make additional testable predictions. The first stage of this model (Figure 4) establishes regions of different V_mem_ within the embryo and provides the input required by the second stage (Figure 5), which shows the dynamics that regulate tumor outcome. The dynamics postulated by the second-stage model require that the left and right sides of the embryo exchange a signal; it is assumed that this alternating signal is implemented, or at least enabled, by coupled oscillations of V_mem_ on the two sides as described in more detail below.

Our two-stage model is based on the observation that large-scale bioelectric cues can provide patterning information during development (Nuccitelli, 2003a,b; McCaig et al., 2005, 2009; Levin, 2012a; Zhao et al., 2012). Let us call the left and right sides of the *Xenopus* embryo A and B, in no particular order. Assume that the two sides exchange a long-range signal that controls proper patterning and subsequent maintenance of order, and that both A and B also respond to cell-autonomous and local signals. We hypothesized that it is an *interaction* between long-range and short-range signals that produces the non-local effects that we see.

If the left and right sides of the embryo are to produce a coherent long-range bioelectric signal, their long-range signaling activity must be coordinated or synchronized. Cells are known, for example, to alternate between hyperpolarization and depolarization with cell cycle (Bregestovski et al., 1992; Arcangeli et al., 1995; Blackiston et al., 2009). If this alternation between hyperpolarization and depolarization was synchronized so that the left side was fully polarized when the right side was fully depolarized and vice-versa, it could produce a coherent long-range signal. The first, “left-right synchronization” stage of our model generates synchronized left and right cell populations from a random initial state. The model assumes a total starting population of 100 cells, corresponding roughly to embryonic cell division seven (2^7^ = 128). Below this number of cells, nearest-neighbor interactions are insufficient to produce global order because too many neighborhoods overlap. The model also assumes an initial left-right bias in the polarization state of the cells [as has been observed experimentally to derive from consistently-asymmetric localization of ion channels on the left and right sides of the 2-cell embryo as it divides (Levin et al., 2002; Qiu et al., 2005; Adams et al., 2006; Morokuma et al., 2008)]. The left-right bias was treated as a free parameter and varied to optimize model behavior. The cells are assumed to occupy a planar array representing the embryonic animal cap epithelium. Each cell is assumed to communicate only with its four nearest neighbors. Each square of the 10 × 10 array (Figure 4) represents one cell at the 100-cell stage; as embryonic cells continue to divide, each square represents the daughter cells in the epithelial “patch” occupied by their progenitor at the 100-cell stage. The left and right boundaries of the planar array are mathematically identified (joined) so that neighborhoods “wrap” around this boundary; the 10 × 10 planar array thus has the topology of a cylinder. Making the upper and lower boundaries join also, to form a spherical topology, was found to have no significant effect on the model's behavior.

The initial random state of the 10 × 10 array was generated by assigning each square of the array two independent integer values: a random “polarization” value between 0 and 255 and a random “depolarization” value between 0 and 255. These random values were then biased by adding a single bias value both to the random “polarization” values of the squares on the left side of the grid and to the random “depolarization” values of the squares on the right side of the grid. At the 100-cell stage, these distinct values represent the potentially-distinct polarization values of distinct cell-membrane patches within a single cell (Wallace, 2007; Adams and Levin, 2013); their sum represents the average V_mem_ of that cell. Following the 100-cell stage, these distinct values also represent the potentially-distinct polarization values of distinct daughter cells within an epithelial patch; their sum represents the average V_mem_ of that patch. The JavaScript Math.random function was used to generate random values. Cell behavior was modeled by a single local rule uniformly implemented by all squares of the array. On each cycle, each square computed the ratio between the total polarization and depolarization values of its four neighbors. If the ratio was greater than or equal to 1, the square multiplied its own polarization value by 1.2 and its own depolarization value by 0.8; if the ratio was less than 1, it did the opposite. These multipliers represent the local response of cells to the average bioelectric state of neighboring cells; cells surrounded by polarized cells become more polarized, while cells surrounded by depolarized cells become less polarized. The values of 1.2 and 0.8 were chosen to produce convergence to a stable state within less than 50 cycles on most model runs; small variations in the values of these multipliers had no significant effect on the qualitative behavior of the model.

This first stage of the model represents the dynamics of bioelectric state change in the early embryo. Its prediction that nearest-neighbor interactions are sufficient to drive convergence to a stable bioelectric state is borne out by timelapse videos using voltage-sensitive fluorescent dyes (Adams and Levin, 2012) in *Xenopus* embryos, which reveal precisely this kind of fluctuation that then settles on a consistent difference in resting potential. Interestingly, the model shows that despite considerable stochastic variability in individual cells' states, such a system consistently converges to a stable state in which the embryo exhibits large regions of identical voltage. In this way, this model for the first time provides an explanation for the observed robustness of bioelectric patterns in development despite the known physiological noise (variability) in individual cells' states.

The model was run until all squares had converged to either fully polarized, defined as a “polarization” value of at least 230 and a “depolarization” value of at most 15, or fully depolarized, defined with the reverse criteria. Replicate model runs that differed only in their initial random states were evaluated on the basis of two criteria: whether the fraction of either polarized or depolarized cells was greater than 65%, and whether more than one region, accounting for “wraps” around the mathematically-identified left and right boundaries, in each state existed, i.e., whether there were “ectopic islands” of polarized or depolarized cells. Due to the model's cylindrical topology, regions of one color, representing one polarization, sometimes “wrap” around the mathematically-identified left and right boundaries, i.e., blue squares appear on the left border and/or red squares appear on the right border. A region was considered “ectopic” only if it comprised one or more squares completely surrounded by squares of the opposite color, or surrounded by squares of the opposite color on three sides if the remaining side formed part of the top or bottom border. The requirement that the number of solutions failing to satisfy either of these criteria is negligible (< 3%) in 100 consecutive runs yields a value of 27% for the initial, 1st-division bias in V_mem_ among the left and right side blastomeres. This prediction of the model is in accordance with data, since direct measurements of voltage states in early embryos, as well as protein and mRNA analyses show consistent and significant biases in the bioelectric states of 2- or 4-cell frog embryos. Decreasing the percent adjustment in polarization and depolarization values on each cycle downward from 20% increases the convergence time for this model without qualitatively changing the results, as does running the model on a 20 × 20 grid (i.e., 400 squares total).

### The two-stage quantitative model accurately predicts the dynamic between V_mem_, GJC, and tumor formation

The existence of bioelectrically-synchronized cell populations on the left and right sides of the embryo that is predicted by the stage-1 model is used by the stage-2, “left-right communication” model to explain the observed results of combining *KRAS^G12D^* with either H7 or Cx26 (Figure 2). Experimental data show that 35% of *KRAS^G12D^*-injected embryos produced tumors; compare this to 44% tumor incidence in embryos injected with *KRAS^G12D^* on both sides (Figure 2). The non-linearity of tumor incidence by itself is an indication of growth control as a result of cross-talk (i.e., a contralateral signal) between sides A and B. Our stage-2 model is based on two primary assumptions. First, we propose that the V_mem_ values of the cells on the left and right sides of the embryo oscillate, in synchrony, with cells on the left polarizing when cells on the right depolarize and vice-versa as discussed above. Normal, non-tumor cells respond to the long-range, synchronized, oscillatory bioelectric signal produced by the contralateral side of the embryo (i.e., to the contralateral signal), but not to the long-range signal that they themselves produce (i.e., the ipsilateral signal). We conceptualize this contralateral signal as a cell-division suppressor; the A and B sides of the embryo are, on this model, alternately signaling to the other side of the embryo to turn the rate of cell division down (Figure 5A), thus maintaining approximate equality of cell-division rates on the two sides. Increased (decreased) contralateral signal is assumed to decrease (increase) tumor formation probability on a % for % basis. Second, we propose that disrupting wild-type GJ with either H7 or Cx26 alters both production of and response to the long-range signal. In particular, we assume that the expression of a dominant negative H7 disrupts response to the contralateral signal (Figures 5C,D) on that side in two ways, by blocking the % for % tumor-suppressing capability of increased contralateral signal, and by causing cells in that same region to increase production of their own (ipsilateral) signal by 50%. We assume the presence of tumor cells on either side disables the increased signal production on that side. The model also requires that tumor cells, but not normal cells, on the contralateral side respond to this increased signal by further suppressing cell division; otherwise the division of normal cells would be over-suppressed. This restriction corresponds biologically to a response saturation on the part of normal cells that is disabled in tumor cells. We model Cx26 as having the opposite effect of increasing response to contralateral signal and decreasing production of ipsilateral signal. We also assume that local disruption of GJC by H7 decreases *KRAS^G12D^* tumor incidence by 20%, while local enhancement of GJC by Cx26 increases tumor incidence by 20%; these local effects are insensitive to contralateral signal.

**Figure 6:**
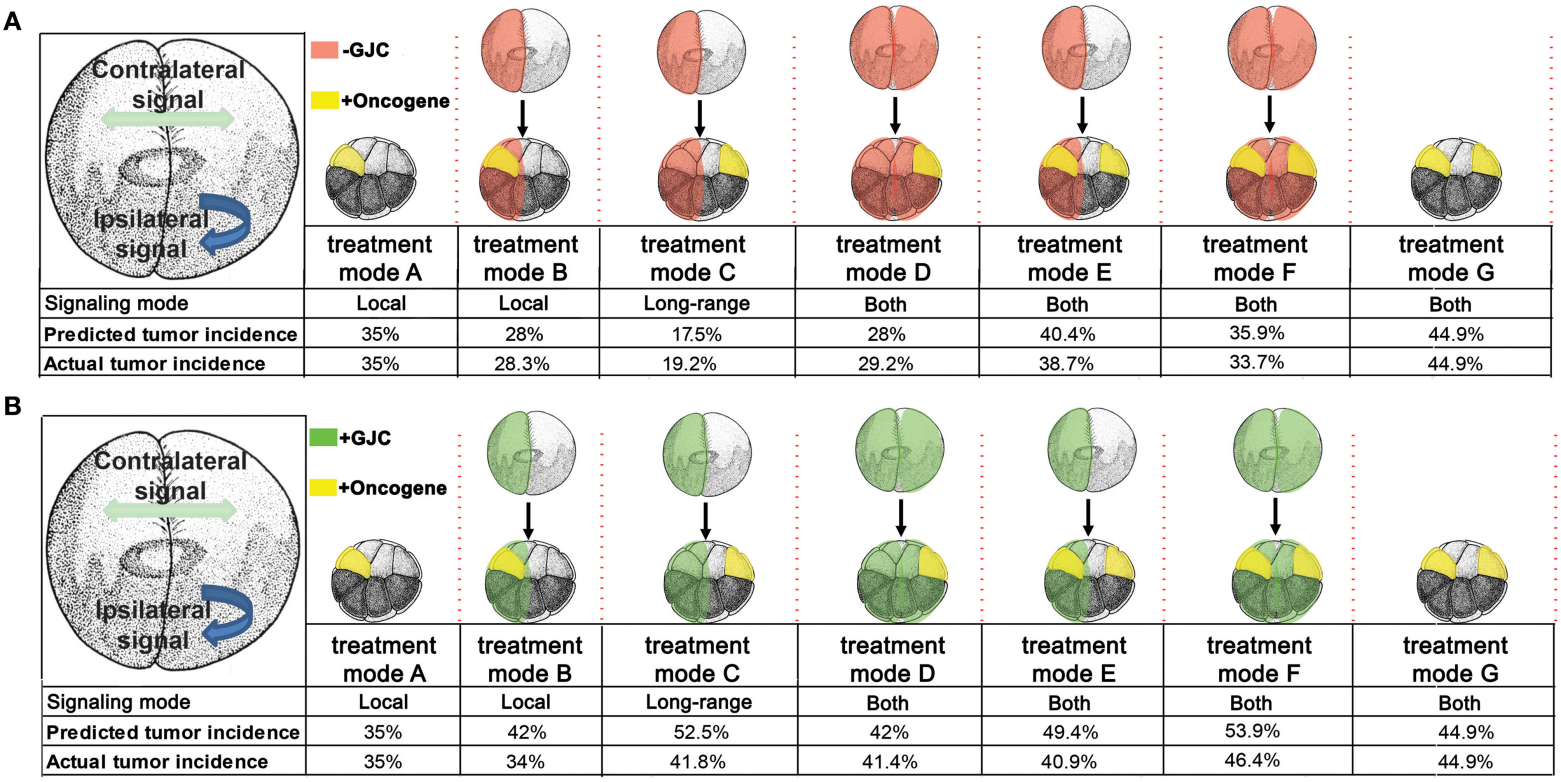
**Predicted tumor incidences that match those of experimental data under GJC perturbations were derived from the stage-2 “left-right communication” model**. In predicting tumor incidences, the left-right communication model implements the following assumptions about cellular responses to *KRAS^G12D^* and H7 **(A)**. *Assumption 1*: embryos have two sides, A and B. During normal embryonic development, sides A and B exchange a handshaking signal that limits cell division. Each side responds to the other side's (i.e. contralateral) signal but not to its own (ipsilateral) signal. A moderately increased or decreased signal is not detrimental to the normal development of unperturbed embryos. *Assumption 2*: experimental data showed 35% of *KRAS^G12D^*-injected embryos produce tumors. *KRAS^G12D^* injections on opposite sides of 16-cell embryos results in 44.9% tumor incidence. The 44% incidence is evenly split between two sides (22.45% incidence per injected side). *Assumption 3*: H7 makes cells deaf to the contralateral signal; in response, they increase production of their own ipsilateral signal, by 50%. *Assumption 4*: Increased (decreased) contralateral signal suppresses (enhances) *KRAS^G12D^* activity on a % for % basis. Ipsilateral H7 directly suppresses oncogenic transformation of *KRAS^G12D^* expressing cells by 20%; contralateral H7 has no direct effect. *Assumption 5*: *KRAS^G12D^* expressing cells disable the increase in signal generation ipsilaterally by interfering with *KRAS^G12D^*-ipsilateral side bioelectric synchronization. Taking into account these five assumptions, the predicted tumor values were calculated for each treatment mode and compared to the observed tumor incidence. The model accurately predicts tumor incidence for every oncogene combination. The *stage-2 “left-right communication” model implements* the following assumptions about cellular responses to *KRAS^G12D^* and Cx26 to account for enhanced (as opposed to disrupted) GJC **(B)**. Assumptions 1 and 2 of the model remain the same as stated in panel **(A)**. *Assumption 3*: Cx26 enhances cells' exposure the contralateral signal; in response, they decrease production of their own ipsilateral signal, by 50%. *Assumption4*: Increased (decreased) contralateral signal suppresses (enhances) *KRAS^G12D^* activity on a % for % basis. Ipsilateral Cx26 directly increases oncogenic transformation of *KRAS^G12D^* cells by 20%; Contralateral Cx26 has no direct effect. While the model predicts the general trend of increased tumorigenesis as a result of enhanced GJC, saturation of tumor incidence around 46% is observed, resulting in higher predictive than actual values.

With these assumptions, it is straightforward to calculate the results expected from the experimental manipulations shown in Figures 2, 3. A single injection of *KRAS^G12D^* on one side of the embryo produces 35% tumors (Figures 2, 3, treatment mode A); this baseline number provides the starting point for calculations. An ipsilateral H7 background decreases cellular response to *KRAS^G12D^* by 20%; hence the expected tumor incidence following Figure 2, treatment mode B is 80% of 35%, i.e., 28%, a prediction error of only 0.3% compared to the observed value (Figure 6A, treatment mode B). The more interesting case is a contralateral H7 background (treatment mode C). Here contralateral H7 causes the contralateral cells to increase signal production by 50%; intuitively, the contralateral cells cannot perceive the signal produced by their neighbors due to disruption of GJC by H7, so they turn up their own signal production to compensate. The *KRAS^G12D^*-ipsilateral side, therefore, receives 50% more contralateral signal. Increased contralateral signal turns *KRAS^G12D^* response down on a % for % basis, so the expected tumor incidence following Figure 2, treatment mode C is 50% of 35%, i.e., 17.5%, a prediction error of 2.2% compared to the observed value. In treatment mode D, both sides of the embryo have an H7 background. Signal production is increased on the side contralateral to *KRAS^G12D^*, but the tumor-suppressing capability of this increased contralateral signal is disrupted by the ipsilateral H7. The situation is, therefore, the same as in treatment mode A; *KRAS^G12D^* activity is only decreased by 20% due to the ipsilateral H7 background. This prediction of 28% tumors has a prediction error of only 1.2% (Figure 6A, treatment mode D). Treatment modes E and F both involve *KRAS^G12D^* injections on both sides of the embryo; here the 44.9% tumor incidence observed with treatment mode G is used as the baseline, i.e., a baseline of 22.45% tumors is assumed on each of the two sides. With *KRAS^G12D^* present on both sides, neither side can increase signal production in response to H7. In treatment mode E, H7 is present on one side, so the predicted tumor incidence is 20% less on that side only; hence the total predicted incidence is (80 × 22.45%) + 22.45% = 40.4%, a prediction error of 1.7%. In treatment mode F, H7 provides 20% tumor suppression on both sides, so the total predicted incidence is (80 × 22.45%) × 2 = 35.9%, a prediction error of 2.2% compared to observations (Figure 6A, treatment mode F). The predictions for Cx26 backgrounds are performed similarly (Figure 6B).

The model assumptions used here effectively specify a control structure (Figure 5E). Each arrow in this structure represents a cellular process regulating response to *KRAS^G12D^*, H7, or Cx26. To objectively test the apparent ability of the model to correctly predict the observed data (Figures 2, 3), we performed a chi-squared test between the model's predicted outcomes (“expected”) and the actual data (“observed”) for the various experimental setups. For the H7 injections, *X*^2^ = 0.547 (*df* = 4), *p* = 0.97, while for the Cx26 injections, *X*^2^ = 5.264 (*df* = 4), *p* = 0.26, thus showing no significant difference between the model's predictions and the experimental data. We conclude that the emergent dynamics of this quantitative model accurately reproduce the complex experimental dataset linking GJC, V_mem_ control, and resulting tumor incidence in various spatial configurations.

### Experimental confirmation of a novel prediction of the model: different outcomes of reagent placement along the left-right vs. dorso-ventral axes

We next tested a novel, surprising prediction of this model that would not have been made without it (by inspection of the experimental data alone): that effectiveness of ion channel injections on tumor suppression will be different depending on which embryonic axis is used to separate the sites of injection.

**Figure 7:**
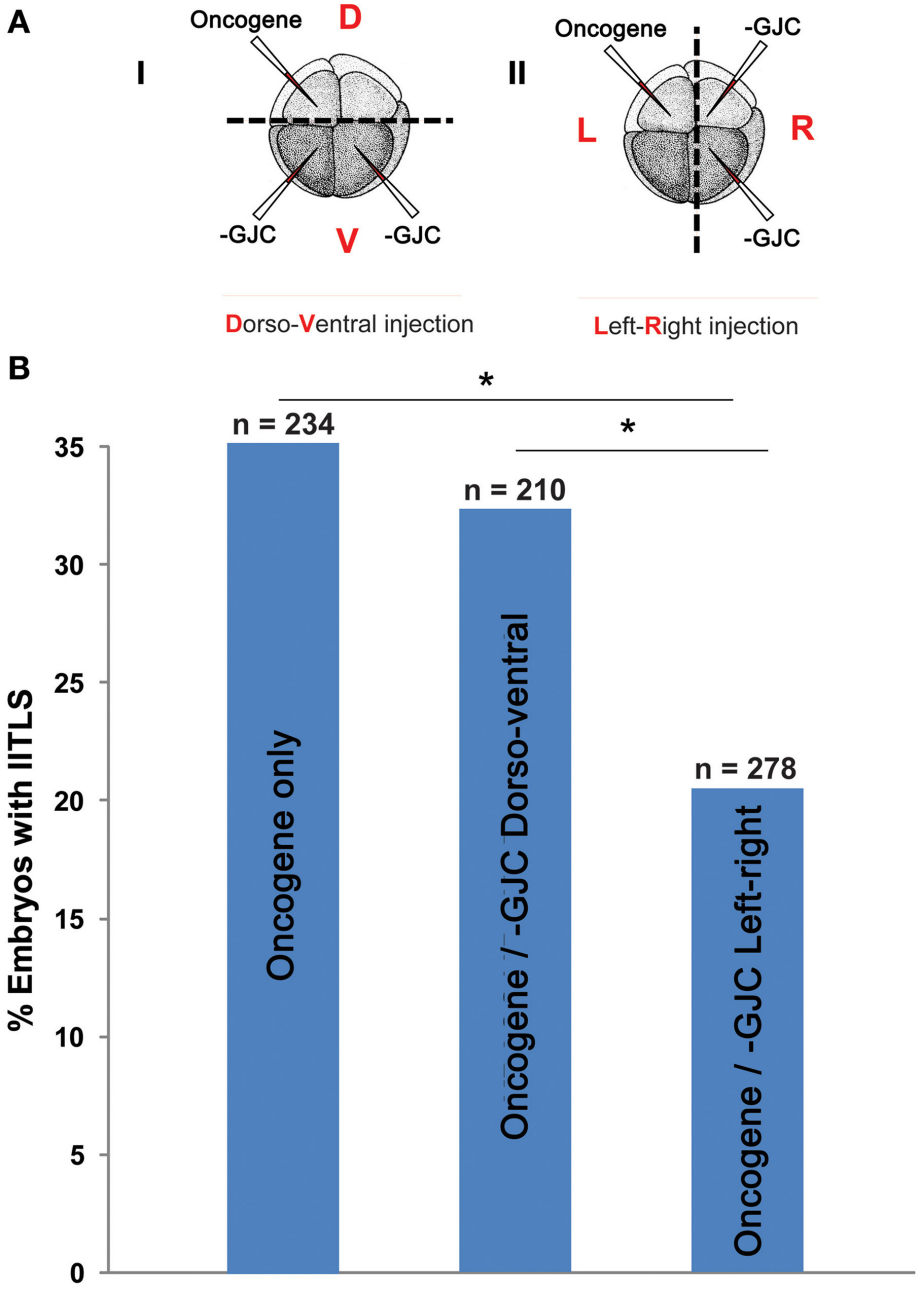
**Testing a unique prediction of our model: tumor incidence is affected by long-range signaling across the left-right axis but not across the dorso-ventral axis. (A)** (i) Embryos were injected with H7 and *KRAS^G12D^*, separately and randomly, in the dorsal and ventral blastomeres. (ii) Embryos were also injected with H7 and *KRAS^G12D^* in the left and right blastomeres, or vise versa. Control embryos injected with *KRAS^G12D^* only displayed a 35% tumor incidence. **(B)** Compared to controls, embryos injected with *KRAS^G12D^* and H7 across the dorsoventral axis did not show a change in tumor incidence (32.4%). Whereas perturbation of GJC communication across the left-right axis significantly lowered tumor incidence down to 20.5% (^*^P < 0.05, *X*^2^ test).

If two neighboring populations of cells are alternating between polarization and depolarization with opposite phase, so that A-side cells are polarized when B-side cells are depolarized and vice-versa, then the cells forming the border between the two populations must maintain a polarization value very near the average for the two populations. These cells, which form the borders between red and blue in Figure 4 can, therefore, be expected to have qualitatively different behavior from cells away from these borders. Therefore, the model also predicts that the ability of GJC reagents to alter the incidence of tumorigenesis should be different depending on whether the opposite sides of the oncogene/H7 axis are oriented with respect to the left-right or dorso-ventral embryonic axes, since the left-right (LR) axis is the one along which gradients of resting potential are known to exist (Levin et al., 2002; Aw et al., 2008). We tested this prediction (Figure 7), and found that indeed the GJC state is most relevant when the opposite-sided distribution of H7-oncogene mRNA coincides with the early embryo's left-right axis. This stage-1 model thus makes unexpected predictions that are supported by experimental test.

## Discussion

### GJ connectivity as a modulator of voltage-dependence of tumor induction

We examined the role of GJC in oncogene-mediated tumorigenesis in *Xenopus* embryos by targeted molecular-genetic modulation. Injection of mRNA constructs that modify gap junctional states in *Xenopus* significantly affected the incidence of tumorigenesis, as detected by increases or decreases in tumor formation after oncogene expression (although the absolute magnitude of the effect of GJ-targeting reagents was limited by the need to use low doses of mRNA to avoid disrupting normal development). Reasonably, the reagent that disrupts GJC and the one that enhances GJC affect tumor incidence in opposite directions. However, we were surprised to see that abrogation of GJC actually suppressed the effects of oncogenes, in contrast to prior suggestions that loss of GJC was a hallmark of incipient cancer (Vine and Bertram, 2002; Sirnes et al., 2012). Interestingly, in addition to confirming *in vivo* the cell-autonomous role inferred for GJCs by cell culture and clinical test data, we found that the greatest impact on tumor incidence occurs when we alter the GJC of cells far away from the tumor. While the microenvironment is increasingly seen to play an important role in cancer (Kenny and Bissell, 2003; Maffini et al., 2005; Hendrix et al., 2007; Kenny et al., 2007; Kasemeier-Kulesa et al., 2008; Tarin, 2012), long-range developmental signals are not yet widely addressed. It should be noted however that two very early workers in the bioelectricity of cancer had previously suggested that such a long-range effect should exist (Burr et al., 1938, 1940; Burr, 1941; Nordenström, 1983).

Targeted expression of wild type connexin Cx26 and dominant negative connexin H7 allowed us to probe how cell:cell communication affects *KRAS^G12D^* activity. Induction of GJC host-wide or non-local to *KRAS^G12D^*-expressing cells led to increases in tumor incidence. Enhanced GJC within *KRAS^G12D^*-expressing cells did not affect tumor incidence. While the increases in tumor incidence seen as a result of GJC induction by Cx26 are significant, the impact of GJC on tumorigenesis was less than expected, partly due to the non-linearity in dose dependence of *KRAS^G12D^* tumor formation. We demonstrated this non-linearity in dose dependence by showing that doubling *KRAS^G12D^* only increases tumor incidence by 9%. Conversely, inhibition of GJC by the dominant negative H7 led to tumor suppression regardless of where H7 was introduced. Both local and host-wide GJC significantly reduced tumor incidence, and long-range disruption of GJC had the most impact on tumor incidence, resulting in 15.8% less embryos with tumor. These results suggest that oncogene-expressing cells utilize the network of GJs between tumors and healthy tissue (Figure 1) to drive tumorigenesis. Most importantly, these data suggest treatment strategies in which oncogene-expressing cells can be junctionally isolated in order to counteract neoplastic transformation.

In contrast to these results, there are significant clinical data implicating GJC as a carcinogenesis suppressor because of its ability to mediate growth control, and that disturbance of GJC between cells is a characteristic of several cancers (Yamasaki, 1990; Rose et al., 1993; Grossman et al., 1994; Hirschi et al., 1996; Yamasaki et al., 1999). However, some tumor cells have been shown to increase expression of functional connexin upon leaving their primary growth sites (Kamibayashi et al., 1995; Zhang et al., 1999). For example, melanoma cells have low levels of Cx26 expression when residing in the epidermal basal layer. However, upon acquiring sustained growth capability, they detach from the epidermis and up-regulate Cx26 expression, which is thought to help them couple with endothelial cells and infiltrate secondary sites (Ito et al., 2000). Overall, the studies presented here highlight scenarios in which enhanced GJC is favorable to tumors and detrimental to the host.

### A quantitative model integrating GJ, voltage, and anatomical axes in tumorigenesis

The results presented here revealed that blocking GJC suppressed tumor formation, while promoting GJC enhanced tumor formation. Even more remarkable was the spatial range over which tumorigenesis could be affected: modulation of GJC at the maximal possible distance from oncogene expressing cells had the most impact on tumor incidence, suggesting a long-range morphogenetic signal that controls tumor growth. In order to formulate a model that quantitatively predicts and explains this puzzling dataset, we undertook a mechanistic/mathematical approach featuring a reasonable set of assumptions.

We propose that H7 disrupts the coordination of polarization-depolarization cycles on the affected side and makes the voltage swings on the affected side bigger; a bigger detected swing suppresses tumor formation on the contralateral side, possibly by affecting the activity of V_mem_ dependent channels. Enhancing GJC with Cx26, in contrast, may disrupt the stability of the boundary separating polarized and depolarized domains. In other words, altering local cell-cell synchrony with GJC-altering reagents may prevent cells from responding in a coordinated way to changes in the polarization states of both their near and distant neighbors, and hence disrupt the oscillating “breathing” pattern required for normal control of cell proliferation. This can be attributed to the disruption of multi-cellular physiological networks established by electrical coupling of gap-junctionally connected cells. One of the interesting aspects of this kind of dynamics is that it is eminently suitable for integration of complex decision-making as would be needed for developmental patterning; for example, the ability of plasmodium organisms to optimize food gathering strategies is currently thought to be implemented by information processing that arises from the integration of oscillatory patterns (Tsuda et al., 2009).

While we do not at this time know the molecular nature of the two signals that implement these dynamics, butyrate and serotonin are good candidates, given previous data on the role of these small signaling molecules in long-range bioelectrical events that regulate cancer (Blackiston et al., 2011; Lobikin et al., 2012; Chernet and Levin, 2013b, 2014). We propose that gap junctions function in this process by regulating ion flow that synchronizes distant cells, enabling global oscillation—the periodic change in the overall electric field within the embryo that provides long-range information exchange. Our model is able to quantitatively account for this surprising dataset and to make new predictions that were verified by experiment. Subsequent work will experimentally test additional predictions of the model. In addition to its implications for control of tumorigenesis, the first phase of the model (establishment of consistent LR gradient from stochastic and highly variable initial V_mem_ cell states) has significant implications for the problem of left-right patterning. If correct, it suggests that the focus of new experiments and theory should be not on stable voltage differences arising directly and immediately from early ion channel localization at 4-cell stage, but on understanding the output of a biased dynamical system operating at 128-cell to blastula stages. It is also significant that our model makes experimentally-verified predictions which functionally link left-right asymmetry and cancer. While connections between these two disparate-seeming fields have been suggested previously (McManus, 1992; Sandson et al., 1992; Wan et al., 2011; Wilting and Hagedorn, 2011; Sauer and Klar, 2012; Veltmaat et al., 2013), this is the first quantitative model of the molecular mechanisms by which these different aspects of pattern regulation are unified.

## Conclusion and perspective

**Figure 8:**
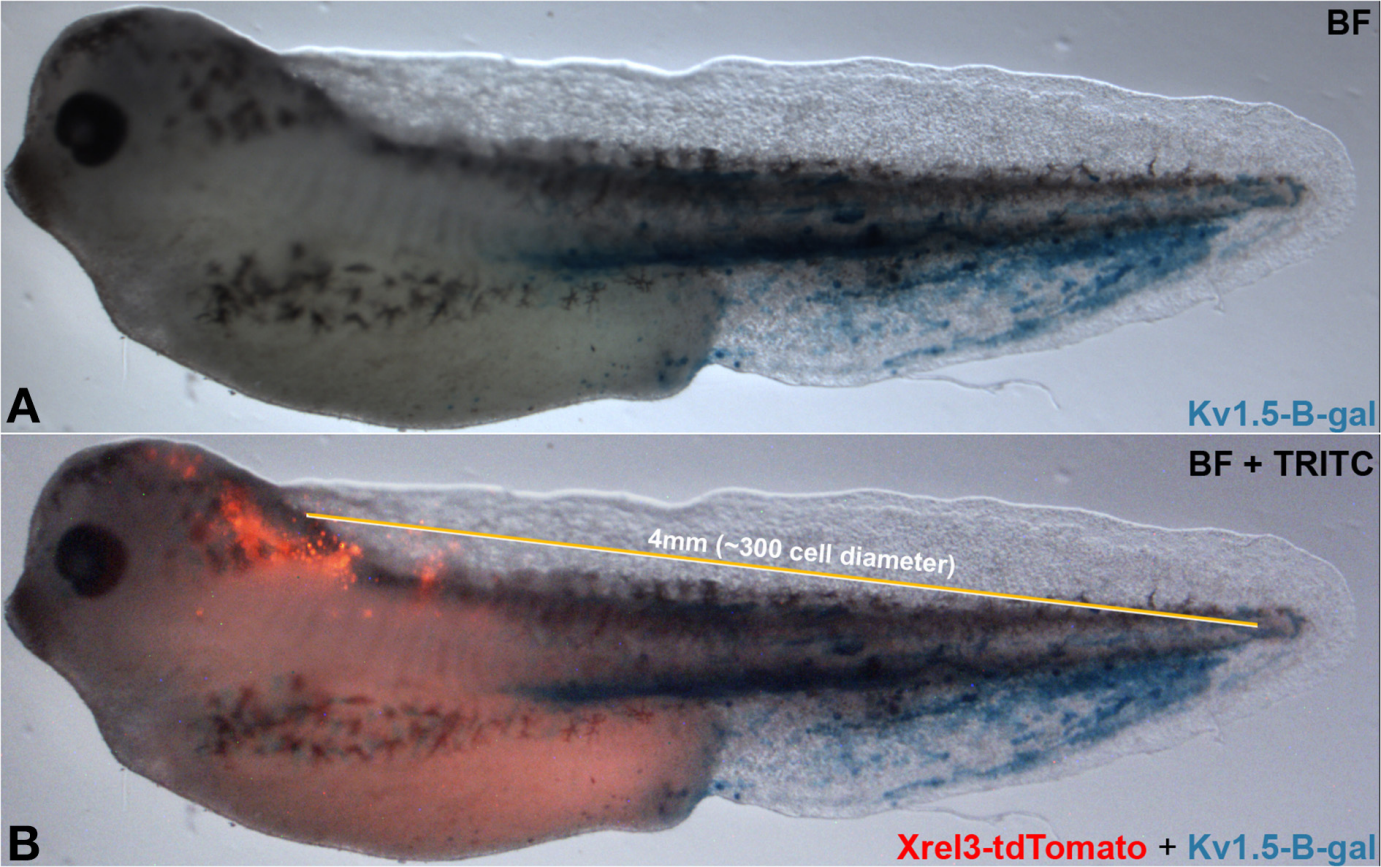
**Long-range signaling in the frog embryo**. Oncogene-expressing cells (red, Xrel3-tdTomato) are prevented from forming tumors by hyperpolarizing channel-expressing cells (Kv 1.5-β-gal) located at a distance. Compare **(A)** without to **(B)** with Xrel3-tdTomato. The ~4 mm distance from the center of the tdTomato signal to the farthest point on the tail expressing β-gal was estimated to be ~300 cell diameter (average single cell diameter of 13.3 μm).

Taken together, our data suggest a complex cross-talk between distant regions of the body that impinge on the stochastic nature of tumorigenesis and physiological dynamics. Additional parameters related to gap junction density include properties such as tissue stiffness, which is known to influence cancer cell behavior and alter cell responses to genetic and epigenetic signals (Bizzarri and Cucina, 2014; Pisanu et al., 2014). Future work will integrate these factors into ever more comprehensive models of tumor formation. While the frog embryo is not considered large by the standards of human medicine (long-range signaling occurs on a scale of from 1.3 to at least 4 mm), it represents about 300 cell diameters (a unit of cell diameter = 13.3 μm)—distinctly non-cell-autonomous (Figure 8). Subsequent work will mechanistically test other predictions of our model, by tracking real-time bioelectrical communication through GJs during tumor induction (a technically very challenging task at the edge of current capability). Our data suggest specific tests of this type of signaling in a mammalian model, and imply that biomedical cancer strategies must consider not only the events of the tumor itself and its microenvironment, but perhaps also signals moving to and from quite remote tissues in the body.

### Conflict of interest statement

The authors declare that the research was conducted in the absence of any commercial or financial relationships that could be construed as a potential conflict of interest.

## References

[B1] Adams D. S. Levin M. (2012). Measuring resting membrane potential using the fluorescent voltage reporters DiBAC4(3) and CC2-DMPE. Cold Spring Harb. Protoc. 2012, 459–464. 10.1101/pdb.prot067702 22474652 PMC4001116

[B2] Adams D. S. Levin M. (2013). Endogenous voltage gradients as mediators of cell-cell communication: strategies for investigating bioelectrical signals during pattern formation. Cell Tissue Res. 352, 95–122. 10.1007/s00441-012-1329-4 22350846 PMC3869965

[B3] Adams D. S. Robinson K. R. Fukumoto T. Yuan S. Albertson R. C. Yelick P.. (2006). Early, H+-V-ATPase-dependent proton flux is necessary for consistent left-right patterning of non-mammalian vertebrates. Development 133, 1657–1671. 10.1242/dev.02341 16554361 PMC3136117

[B4] Arcangeli A. Bianchi L. Becchetti A. Faravelli L. Coronnello M. Mini E.. (1995). A novel inward-rectifying K+ current with a cell-cycle dependence governs the resting potential of mammalian neuroblastoma cells. J. Physiol. 489, 455–471. 10.1113/jphysiol.1995.sp021065 8847640 PMC1156772

[B5] Arcangeli A. Crociani O. Lastraioli E. Masi A. Pillozzi S. Becchetti A. (2009). Targeting ion channels in cancer: a novel frontier in antineoplastic therapy. Curr. Med. Chem. 16, 66–93. 10.2174/092986709787002835 19149563

[B6] Arcangeli A. Pillozzi S. Becchetti A. (2012). Targeting ion channels in leukemias: a new challenge for treatment. Curr. Med. Chem. 19, 683–696. 10.2174/092986712798992093 22204341

[B8] Aw S. Adams D. S. Qiu D. Levin M. (2008). H,K-ATPase protein localization and Kir4.1 function reveal concordance of three axes during early determination of left-right asymmetry. Mech. Dev. 125, 353–372. 10.1016/j.mod.2007.10.011 18160269 PMC2346612

[B9] Barrio L. C. Capel J. Jarillo J. A. Castro C. Revilla A. (1997). Species-specific voltage-gating properties of connexin-45 junctions expressed in Xenopus oocytes. Biophys. J. 73, 757–769. 10.1016/S0006-3495(97)78108-6 9251792 PMC1180972

[B10] Bennett M. V. (1997). Gap junctions as electrical synapses. J. Neurocytol. 26, 349–366. 10.1023/A:1018560803261 9278865

[B12] Bissell M. J. Hines W. C. (2011). Why don't we get more cancer? A proposed role of the microenvironment in restraining cancer progression. Nat. Med. 17, 320–329. 10.1038/nm.2328 21383745 PMC3569482

[B13] Bizzarri M. Cucina A. (2014). Tumor and the microenvironment: a chance to reframe the paradigm of carcinogenesis? Biomed Res. Int. 2014:934038. 10.1155/2014/934038 25013812 PMC4075186

[B14] Blackiston D. Adams D. S. Lemire J. M. Lobikin M. Levin M. (2011). Transmembrane potential of GlyCl-expressing instructor cells induces a neoplastic-like conversion of melanocytes via a serotonergic pathway. Dis. Model Mech. 4, 67–85. 10.1242/dmm.005561 20959630 PMC3008964

[B15] Blackiston D. J. McLaughlin K. A. Levin M. (2009). Bioelectric controls of cell proliferation: ion channels, membrane voltage and the cell cycle. Cell Cycle 8, 3519–3528. 10.4161/cc.8.21.9888 19823012 PMC2862582

[B16] Bregestovski P. Medina I. Goyda E. (1992). Regulation of potassium conductance in the cellular membrane at early embryogenesis. J. Physiol. Paris 86, 109–115. 10.1016/S0928-4257(05)80014-2 1343588

[B17] Bruzzone R. White T. W. Goodenough D. A. (1996a). The cellular Internet: on-line with connexins. Bioessays 18, 709–718. 8831287 10.1002/bies.950180906

[B18] Bruzzone R. White T. W. Paul D. L. (1996b). Connections with connexins: the molecular basis of direct intercellular signaling. Eur. J. Biochem. 238, 1–27. 8665925 10.1111/j.1432-1033.1996.0001q.x

[B19] Burr H. S. (1941). Changes in the field properties of mice with transplanted tumors. Yale J. Biol. Med. 13, 783–788. 21433982 PMC2602583

[B20] Burr H. S. Smith G. M. Strong L. C. (1940). Electrometric studies of tumors in mice induced by the external application of benzpyrene. Yale J. Biol. Med. 12, 711–717. 21433919 PMC2602452

[B21] Burr H. S. Strong L. C. Smith G. M. (1938). Bio-Electric Correlates of Methylcolanthrene-Induced Tumors in mice. Yale J. Biol. Med. 10, 539–544. 21433788 PMC2601931

[B22] Cao F. Eckert R. Elfgang C. Nitsche J. M. Snyder S. A. H-ulser D. F.. (1998). A quantitative analysis of connexin-specific permeability differences of gap junctions expressed in HeLa transfectants and Xenopus oocytes. J. Cell Sci. 111(Pt 1), 31–43. 9394010 10.1242/jcs.111.1.31

[B23] Chernet B. Levin M. (2013a). Endogenous voltage potentials and the microenvironment: bioelectric signals that reveal, induce and normalize cancer. J. Clin. Exp. Oncol. (Suppl 1) S1-002. 10.4172/2324-9110.S1-002 25525610 PMC4267524

[B24] Chernet B. T. Levin M. (2013b). Transmembrane voltage potential is an essential cellular parameter for the detection and control of tumor development in a Xenopus model. Dis. Model Mech. 6, 595–607. 10.1242/dmm.010835 23471912 PMC3634644

[B25] Chernet B. T. Levin M. (2014). Transmembrane voltage potential of somatic cells controls oncogene-mediated tumorigenesis at long-range. Oncotarget 5, 3287–3306. 24830454 10.18632/oncotarget.1935 PMC4102810

[B26] Clark W. H. Jr. (1995). The nature of cancer: morphogenesis and progressive (self)-disorganization in neoplastic development and progression. Acta Oncol. 34, 3–21. 10.3109/02841869509093632 7865232

[B27] Dale L. Slack J. (1987). Fate map for the 32-cell stage of *Xenopus laevis*. Development 100, 279–296. 3665770 10.1242/dev.99.4.527

[B29] Dean M. (1998). Cancer as a complex developmental disorder–nineteenth Cornelius P. Rhoads Memorial Award Lecture. Cancer Res. 58, 5633–5636. 9865711

[B30] Diss J. K. Stewart D. Pani F. Foster C. S. Walker M. M. Patel A.. (2005). A potential novel marker for human prostate cancer: voltage-gated sodium channel expression *in vivo*. Prostate Cancer Prostatic Dis. 8, 266–273. 10.1038/sj.pcan.4500796 16088330

[B31] Donahue H. J. Saunders M. M. Li Z. Mastro A. M. Gay C. V. Welch D. R. (2003). A potential role for gap junctions in breast cancer metastasis to bone. J. Musculoskelet. Neuronal Interact. 3, 156–161. 15758356

[B32] Duflot-Dancer A. Mesnil M. Yamasaki H. (1997). Dominant-negative abrogation of connexin-mediated cell growth control by mutant connexin genes. Oncogene 15, 2151–2158. 10.1038/sj.onc.1201393 9393973

[B33] Elzarrad M. K. Haroon A. Willecke K. Dobrowolski R. Gillespie M. N. Al-Mehdi A. B. (2008). Connexin-43 upregulation in micrometastases and tumor vasculature and its role in tumor cell attachment to pulmonary endothelium. BMC Med. 6:20. 10.1186/1741-7015-6-20 18647409 PMC2492868

[B34] Ewart J. L. Cohen M. F. Meyer R. A. Huang G. Y. Wessels A. Gourdie R. G.. (1997). Heart and neural tube defects in transgenic mice overexpressing the Cx43 gap junction gene. Development 124, 1281–1292. 9118799 10.1242/dev.124.7.1281

[B35] Fraser S. P. Diss J. K. Chioni A. M. Mycielska M. E. Pan H. Yamaci R. F.. (2005). Voltage-gated sodium channel expression and potentiation of human breast cancer metastasis. Clin. Cancer Res. 11, 5381–5389. 10.1158/1078-0432.CCR-05-0327 16061851

[B36] Gee J. Tanaka M. Grossman H. B. (2003). Connexin 26 is abnormally expressed in bladder cancer. J. Urol. 169, 1135–1137. 10.1097/01.ju.0000041954.91331.df 12576868

[B37] Goldberg G. S. Valiunas V. Brink P. R. (2004). Selective permeability of gap junction channels. Biochim. Biophys. Acta 1662, 96–101. 10.1016/j.bbamem.2003.11.022 15033581

[B38] Goodenough D. A. Goliger J. A. Paul D. L. (1996). Connexins, connexons, and intercellular communication. Annu. Rev. Biochem. 65, 475–502. 10.1146/annurev.bi.65.070196.002355 8811187

[B39] Grossman H. B. Liebert M. Lee I. W. Lee S. W. (1994). Decreased connexin expression and intercellular communication in human bladder cancer cells. Cancer Res. 54, 3062–3065. 8187096

[B40] Haass N. K. Ripperger D. Wladykowski E. Dawson P. Gimotty P. A. Blome C.. (2010). Melanoma progression exhibits a significant impact on connexin expression patterns in the epidermal tumor microenvironment. Histochem. Cell Biol. 133, 113–124. 10.1007/s00418-009-0654-5 19844737

[B41] Hendrix M. J. Seftor E. A. Seftor R. E. Kasemeier-Kulesa J. Kulesa P. M. Postovit L. M. (2007). Reprogramming metastatic tumour cells with embryonic microenvironments. Nat. Rev. Cancer 7, 246–255. 10.1038/nrc2108 17384580

[B42] Hirschi K. K. Xu C. E. Tsukamoto T. Sager R. (1996). Gap junction genes Cx26 and Cx43 individually suppress the cancer phenotype of human mammary carcinoma cells and restore differentiation potential. Cell Growth Differ. 7, 861–870. 8809403

[B43] House C. D. Vaske C. J. Schwartz A. Obias V. Frank B. Luu T.. (2010). Voltage-gated Na+ channel SCN5A is a key regulator of a gene transcriptional network that controls colon cancer invasion. Cancer Res. 70, 6957–6967. 10.1158/0008-5472.CAN-10-1169 20651255 PMC2936697

[B44] Ito A. Katoh F. Kataoka T. R. Okada M. Tsubota N. Asada H.. (2000). A role for heterologous gap junctions between melanoma and endothelial cells in metastasis. J. Clin. Invest. 105, 1189–1197. 10.1172/JCI8257 10791993 PMC315440

[B45] Jongsma H. J. Wilders R. (2000). Gap junctions in cardiovascular disease. Circ. Res. 86, 1193–1197. 10.1161/01.RES.86.12.1193 10864907

[B46] Kamibayashi Y. Oyamada Y. Mori M. Oyamada M. (1995). Aberrant expression of gap junction proteins (connexins) is associated with tumor progression during multistage mouse skin carcinogenesis *in vivo*. Carcinogenesis 16, 1287–1297. 10.1093/carcin/16.6.1287 7788845

[B47] Kasemeier-Kulesa J. C. Teddy J. M. Postovit L. M. Seftor E. A. Seftor R. E. Hendrix M. J.. (2008). Reprogramming multipotent tumor cells with the embryonic neural crest microenvironment. Dev. Dyn. 237, 2657–2666. 10.1002/dvdy.21613 18629870 PMC2570047

[B48] Kenny P. A. Bissell M. J. (2003). Tumor reversion: correction of malignant behavior by microenvironmental cues. International journal of cancer. J. Int. Cancer 107, 688–695. 10.1002/ijc.11491 14566816 PMC2933180

[B49] Kenny P. A. Lee G. Y. Bissell M. J. (2007). Targeting the tumor microenvironment. Front. Biosci. 12, 3468–3474. 10.2741/2327 17485314 PMC2841020

[B50] King T. J. Bertram J. S. (2005). Connexins as targets for cancer chemoprevention and chemotherapy. Biochim. Biophys. Acta 1719, 146–160. 10.1016/j.bbamem.2005.08.012 16263076

[B52] Krysko D. V. Leybaert L. Vandenabeele P. D'Herde K. (2005). Gap junctions and the propagation of cell survival and cell death signals. Apoptosis 10, 459–469. 10.1007/s10495-005-1875-2 15909108

[B53] Le X. Langenau D. M. Keefe M. D. Kutok J. L. Neuberg D. S. Zon L. I. (2007). Heat shock-inducible Cre/Lox approaches to induce diverse types of tumors and hyperplasia in transgenic zebrafish. Proc. Natl. Acad. Sci. U.S.A. 104, 9410–9415. 10.1073/pnas.0611302104 17517602 PMC1890508

[B54] Lee J. R. Derosa A. M. White T. W. (2009). Connexin mutations causing skin disease and deafness increase hemichannel activity and cell death when expressed in Xenopus oocytes. J. Invest. Dermatol. 129, 870–878. 10.1038/jid.2008.335 18987669 PMC6463483

[B55] Levin M. (2002). Isolation and community: a review of the role of gap-junctional communication in embryonic patterning. J. Membr. Biol. 185, 177–192. 10.1007/s00232-001-0129-7 11891576

[B56] Levin M. (2007). Gap junctional communication in morphogenesis. Prog. Biophys. Mol. Biol. 94, 186–206. 10.1016/j.pbiomolbio.2007.03.005 17481700 PMC2292839

[B57] Levin M. (2011). The wisdom of the body: future techniques and approaches to morphogenetic fields in regenerative medicine, developmental biology and cancer. Regen. Med. 6, 667–673. 10.2217/rme.11.69 22050517

[B58] Levin M. (2012a). Molecular bioelectricity in developmental biology: new tools and recent discoveries: control of cell behavior and pattern formation by transmembrane potential gradients. Bioessays 34, 205–217. 10.1002/bies.201100136 22237730 PMC3430077

[B59] Levin M. (2012b). Morphogenetic fields in embryogenesis, regeneration, and cancer: non-local control of complex patterning. Biosystems 109, 243–261. 10.1016/j.biosystems.2012.04.005 22542702 PMC3413735

[B60] Levin M. (2013). Reprogramming cells and tissue patterning via bioelectrical pathways: molecular mechanisms and biomedical opportunities. Wiley Interdiscip. Rev. Syst. Biol. Med. 5, 657–676. 10.1002/wsbm.1236 23897652 PMC3841289

[B61] Levin M. (2014). Endogenous bioelectrical networks store non-genetic patterning information during development and regeneration. J. Physiol. 592, 2295–2305. 10.1113/jphysiol.2014.271940 24882814 PMC4048089

[B62] Levin M. Mercola M. (1998). Gap junctions are involved in the early generation of left-right asymmetry. Dev. Biol. 203, 90–105. 10.1006/dbio.1998.9024 9806775

[B63] Levin M. Mercola M. (1999). Gap junction-mediated transfer of left-right patterning signals in the early chick blastoderm is upstream of Shh asymmetry in the node. Development 126, 4703–4714. 10518488 10.1242/dev.126.21.4703

[B64] Levin M. Thorlin T. Robinson K. R. Nogi T. Mercola M. (2002). Asymmetries in H+/K+-ATPase and cell membrane potentials comprise a very early step in left-right patterning. Cell 111, 77–89. 10.1016/S0092-8674(02)00939-X 12372302

[B65] Lewalle J. M. Cataldo D. Bajou K. Lambert C. A. Foidart J. M. (1998). Endothelial cell intracellular Ca2+ concentration is increased upon breast tumor cell contact and mediates tumor cell transendothelial migration. Clin. Exp. Metastasis 16, 21–29. 10.1023/A:1006555800862 9502074

[B66] Lobikin M. Chernet B. Lobo D. Levin M. (2012). Resting potential, oncogene-induced tumorigenesis, and metastasis: the bioelectric basis of cancer *in vivo*. Phys. Biol. 9:065002. 10.1088/1478-3975/9/6/065002 23196890 PMC3528107

[B68] Loewenstein W. R. (1979). Junctional intercellular communication and the control of growth. Biochim. Biophys. Acta. 560, 1–65. 216404 10.1016/0304-419x(79)90002-7

[B69] Loewenstein W. R. (1981). Junctional intercellular communication: the cell-to-cell membrane channel. Physiol. Rev. 61, 829–913. 6270711 10.1152/physrev.1981.61.4.829

[B70] Loewenstein W. R. Kanno Y. (1966). Intercellular communication and the control of tissue growth: lack of communication between cancer cells. Nature 209, 1248–1249. 10.1038/2091248a0 5956321

[B71] Maffini M. V. Calabro J. M. Soto A. M. Sonnenschein C. (2005). Stromal regulation of neoplastic development: age-dependent normalization of neoplastic mammary cells by mammary stroma. Am. J. Pathol. 167, 1405–1410. 10.1016/S0002-9440(10)61227-8 16251424 PMC1603788

[B72] Magnon C. Hall S. J. Lin J. Xue X. Gerber L. Freedland S. J.. (2013). Autonomic nerve development contributes to prostate cancer progression. Science 341:1236361. 10.1126/science.1236361 23846904

[B73] Marongiu F. Doratiotto S. Sini M. Serra M. P. Laconi E. (2012). Cancer as a disease of tissue pattern formation. Prog. Histochem. Cytochem. 47, 175–207. 10.1016/j.proghi.2012.08.001 22985795

[B74] McCaig C. D. Rajnicek A. M. Song B. Zhao M. (2005). Controlling cell behavior electrically: current views and future potential. Physiol. Rev. 85, 943–978. 10.1152/physrev.00020.2004 15987799

[B75] McCaig C. D. Song B. Rajnicek A. M. (2009). Electrical dimensions in cell science. J. Cell Sci. 122, 4267–4276. 10.1242/jcs.023564 19923270

[B76] McManus I. C. (1992). Reversed cerebral asymmetry and breast cancer. Lancet 339, 523–524 10.1016/0140-6736(92)90577-P 1349077

[B77] Mesnil M. Crespin S. Avanzo J. L. Zaidan-Dagli M. L. (2005). Defective gap junctional intercellular communication in the carcinogenic process. Biochim. Biophys. Acta 1719, 125–145. 10.1016/j.bbamem.2005.11.004 16359943

[B78] Mesnil M. Piccoli C. Yamasaki H. (1997). A tumor suppressor gene, Cx26, also mediates the bystander effect in HeLa cells. Cancer Res. 57, 2929–2932. 9230203

[B79] Moody S. A. Kline M. J. (1990). Segregation of fate during cleavage of frog (*Xenopus laevis*) blastomeres. Anat. Embryol. 182, 347–362. 10.1007/BF02433495 2252221

[B80] Morokuma J. Blackiston D. Levin M. (2008). KCNQ1 and KCNE1 K+ channel components are involved in early left-right patterning in *Xenopus laevis* embryos. Cell. Physiol. Biochem. 21, 357–372. 10.1159/000129628 18453744 PMC3632048

[B81] Naoi Y. Miyoshi Y. Taguchi T. Kim S. J. Arai T. Tamaki Y.. (2007). Connexin26 expression is associated with lymphatic vessel invasion and poor prognosis in human breast cancer. Breast Cancer Res. Treat. 106, 11–17. 10.1007/s10549-006-9465-8 17203385

[B82] Nieuwkoop P. D. Faber J. (1967). Normal Table of Xenopus Laevis (Daudin). Amsterdam: North-Holland Publishing Company.

[B83] Nordenström B. (1983). Biologically Closed Electric Circuits: Clinical, Experimental, and Theoretical Evidence for an Additional Circulatory System. Stockholm: Nordic Medical Publications.

[B84] Nuccitelli R. (2003a). Endogenous electric fields in embryos during development, regeneration and wound healing. Radiat. Prot. Dosimetry. 106, 375–383. 10.1093/oxfordjournals.rpd.a006375 14690282

[B85] Nuccitelli R. (2003b). A role for endogenous electric fields in wound healing. Curr. Top. Dev. Biol. 58, 1–26. 10.1016/S0070-2153(03)58001-2 14711011

[B86] Oviedo N. J. Morokuma J. Walentek P. Kema I. P. Gu M. B. Ahn J. M.. (2010). Long-range neural and gap junction protein-mediated cues control polarity during planarian regeneration. Dev. Biol. 339, 188–199. 10.1016/j.ydbio.2009.12.012 20026026 PMC2823934

[B87] Palacios-Prado N. Bukauskas F. F. (2009). Heterotypic gap junction channels as voltage-sensitive valves for intercellular signaling. Proc. Natl. Acad. Sci. U.S.A. 106, 14855–14860. 10.1073/pnas.0901923106 19706392 PMC2736430

[B88] Pardo L. A. Stuhmer W. (2014). The roles of K(+) channels in cancer. Nat. Rev. Cancer 14, 39–48. 10.1038/nrc3635 24336491

[B89] Paul D. L. Yu K. Bruzzone R. Gimlich R. L. Goodenough D. A. (1995). Expression of a dominant negative inhibitor of intercellular communication in the early Xenopus embryo causes delamination and extrusion of cells. Development 121, 371–381. 7768179 10.1242/dev.121.2.371

[B90] Pawlowski A. Weddell G. (1967). Induction of tumours in denervated skin. Nature 213, 1234–1236 10.1038/2131234a0

[B91] Pereda A. E. Curti S. Hoge G. Cachope R. Flores C. E. Rash J. E. (2013). Gap junction-mediated electrical transmission: regulatory mechanisms and plasticity. Biochim. Biophys. Acta 1828, 134–146. 10.1016/j.bbamem.2012.05.026 22659675 PMC3437247

[B92] Pierce G. B. Speers W. C. (1988). Tumors as caricatures of the process of tissue renewal: prospects for therapy by directing differentiation. Cancer Res. 48, 1996–2004. 2450643

[B93] Pisanu M. E. Noto A. De Vitis C. Masiello M. G. Coluccia P. Proietti S.. (2014). Lung cancer stem cell lose their stemness default state after exposure to microgravity. Biomed Res. Int. 2014:470253. 10.1155/2014/470253 25276790 PMC4170742

[B94] Qiu D. Cheng S. M. Wozniak L. McSweeney M. Perrone E. Levin M. (2005). Localization and loss-of-function implicates ciliary proteins in early, cytoplasmic roles in left-right asymmetry. Dev. Dyn. 234, 176–189. 10.1002/dvdy.20509 16059906

[B95] Rabionet R. Gasparini P. Estivill X. (2000). Molecular genetics of hearing impairment due to mutations in gap junction genes encoding beta connexins. Hum. Mut. 16, 190–202. 10.1002/1098-1004(200009)16:3<190::AID-HUMU2>3.0.CO;2-I 10980526

[B96] Rash J. E. Curti S. Vanderpool K. G. Kamasawa N. Nannapaneni S. Palacios-Prado N.. (2013). Molecular and functional asymmetry at a vertebrate electrical synapse. Neuron 79, 957–969. 10.1016/j.neuron.2013.06.037 24012008 PMC4020187

[B97] Rose B. Mehta P. P. Loewenstein W. R. (1993). Gap-junction protein gene suppresses tumorigenicity. Carcinogenesis 14, 1073–1075. 10.1093/carcin/14.5.1073 8389252

[B98] Rose S. M. Wallingford H. M. (1948). Transformation of renal tumors of frogs to normal tissues in regenerating limbs of salamanders. Science 107, 457. 18938459

[B99] Rubin H. (2006). What keeps cells in tissues behaving normally in the face of myriad mutations? Bioessays 28, 515–524. 10.1002/bies.20403 16615084

[B100] Ruch R. J. Trosko J. E. (2001). Gap-junction communication in chemical carcinogenesis. Drug Metab. Rev. 33, 117–124. 10.1081/DMR-100000137 11270660

[B101] Saito-Katsuragi M. Asada H. Niizeki H. Katoh F. Masuzawa M. Tsutsumi M.. (2007). Role for connexin 26 in metastasis of human malignant melanoma: communication between melanoma and endothelial cells via connexin 26. Cancer 110, 1162–1172. 10.1002/cncr.22894 17614106

[B102] Sandson T. A. Wen P. Y. LeMay M. (1992). Reversed cerebral asymmetry in women with breast cancer. Lancet 339, 523–524. 10.1016/0140-6736(92)90341-Y 1346881

[B103] Sauer S. Klar A. J. (2012). Left-right symmetry breaking in mice by left-right dynein may occur via a biased chromatid segregation mechanism, without directly involving the Nodal gene. Front. Oncol. 2:166. 10.3389/fonc.2012.00166 23316472 PMC3540932

[B104] Scharrer B. (1953). Insect tumors induced by nerve severance: incidence and mortality. Cancer Res. 13, 73–76. 13032953

[B105] Sirnes S. Bruun J. Kolberg M. Kjenseth A. Lind G. E. Svindland A.. (2012). Connexin43 acts as a colorectal cancer tumor suppressor and predicts disease outcome. International journal of cancer. J. Int. Cancer 131, 570–581. 10.1002/ijc.26392 21866551

[B106] Sive H. Grainger R. M. Harland R. M. (2000). Early Development of Xenopus Laevis: A Laboratory Manual. New York, NY: Cold Spring Harbor Laboratory Press.

[B107] Soroceanu L. Manning T. J. Jr. Sontheimer H. (2001). Reduced expression of connexin-43 and functional gap junction coupling in human gliomas. GLIA 33, 107–117. 10.1002/1098-1136(200102)33:2<107::AID-GLIA1010>3.0.CO;2-4 11180508

[B108] Stoletov K. Strnadel J. Zardouzian E. Momiyama M. Park F. D. Kelber J. A.. (2013). Role of connexins in metastatic breast cancer and melanoma brain colonization. J. Cell Sci. 126, 904–913. 10.1242/jcs.112748 23321642 PMC3625812

[B109] Swenson K. I. Jordan J. R. Beyer E. C. Paul D. L. (1989). Formation of gap junctions by expression of connexins in Xenopus oocyte pairs. Cell 57, 145–155. 10.1016/0092-8674(89)90180-3 2467743

[B110] Talbot J. Brion R. Picarda G. Amiaud J. Chesneau J. Bougras G.. (2013). Loss of connexin43 expression in Ewing's sarcoma cells favors the development of the primary tumor and the associated bone osteolysis. Biochim. Biophys. Acta 1832, 553–564. 10.1016/j.bbadis.2013.01.001 23313578

[B111] Tarin D. (2012). Clinical and biological implications of the tumor microenvironment. Cancer Microenviron. 5, 95–112. 10.1007/s12307-012-0099-6 22782446 PMC3399064

[B112] Temme A. Buchmann A. Gabriel H. D. Nelles E. Schwarz M. Willecke K. (1997). High incidence of spontaneous and chemically induced liver tumors in mice deficient for connexin32. Curr. Biol. 7, 713–716. 10.1016/S0960-9822(06)00302-2 9285723

[B113] Than B. L. Goos J. A. Sarver A. L. O'Sullivan M. G. Rod A. Starr T. K.. (2014). The role of KCNQ1 in mouse and human gastrointestinal cancers. Oncogene 33, 3861–3868. 10.1038/onc.2013.350 23975432 PMC3935979

[B114] Trosko J. E. (2005). The role of stem cells and gap junctions as targets for cancer chemoprevention and chemotherapy. Biomed. Pharmacother. 59(Suppl. 2), S326–S331. 10.1016/S0753-3322(05)80065-4 16507402

[B115] Tseng A. Levin M. (2013). Cracking the bioelectric code: probing endogenous ionic controls of pattern formation. Commun. Integr. Biol. 6, 1–8. 10.4161/cib.22595 23802040 PMC3689572

[B116] Tsonis P. A. (1987). Embryogenesis and carcinogenesis: order and disorder. Anticancer Res. 7, 617–623. 3310849

[B117] Tsuda S. Artmann S. Zauner K.-P. (2009). The phi-bot:a robot controlled by a slime, mould, in Artificial Life Models in Hardware, eds Adamatzky A. Komosinski M. (London: Springer), 213–232.

[B118] Vega A. V. Avila G. Matthews G. (2013). Interaction between the transcriptional corepressor Sin3B and voltage-gated sodium channels modulates functional channel expression. Sci. Rep. 3:2809. 10.1038/srep02809 24077057 PMC3786298

[B119] Veltmaat J. M. Ramsdell A. F. Sterneck E. (2013). Positional variations in mammary gland development and cancer. J. Mammary Gland Biol. Neoplasia 18, 179–188. 10.1007/s10911-013-9287-3 23666389 PMC3691492

[B120] Vine A. L. Bertram J. S. (2002). Cancer chemoprevention by connexins. Cancer Metastasis Rev. 21, 199–216. 10.1023/A:1021250624933 12549761

[B121] Wallace R. (2007). Neural membrane microdomains as computational systems: toward molecular modeling in the study of neural disease. Biosystems 87, 20–30. 10.1016/j.biosystems.2006.02.012 16650927

[B122] Wan L. Q. Ronaldson K. Park M. Taylor G. Zhang Y. Gimble J. M.. (2011). Micropatterned mammalian cells exhibit phenotype-specific left-right asymmetry. Proc. Natl. Acad. Sci. U.S.A. 108, 12295–12300. 10.1073/pnas.1103834108 21709270 PMC3145729

[B123] Warner A. (1992). Gap junctions in development–a perspective. Semin. Cell Biol. 3, 81–91. 10.1016/S1043-4682(10)80009-1 1320432

[B124] Wilting J. Hagedorn M. (2011). Left-right asymmetry in embryonic development and breast cancer: common molecular determinants? Curr. Med. Chem. 18, 5519–5527. 10.2174/092986711798347252 22172062

[B125] Wong R. C. Pera M. F. Pebay A. (2008). Role of gap junctions in embryonic and somatic stem cells. Stem Cell Rev. 4, 283–292. 10.1007/s12015-008-9038-9 18704771

[B126] Yamasaki H. (1990). Gap junctional intercellular communication and carcinogenesis. Carcinogenesis 11, 1051–1058. 10.1093/carcin/11.7.1051 2197009

[B127] Yamasaki H. Krutovskikh V. Mesnil M. Tanaka T. Zaidan-Dagli M. Omori Y. (1999). Role of connexin (gap junction) genes in cell growth control and carcinogenesis. Comptes Rendus de l Acad. des Sci. 322, 151–159. 10.1016/S0764-4469(99)80038-9 10196667

[B128] Yamasaki H. Mesnil M. Omori Y. Mironov N. Krutovskikh V. (1995). Intercellular communication and carcinogenesis. Mut. Res. 333, 181–188. 10.1016/0027-5107(95)00144-1 8538626

[B129] Yang M. Brackenbury W. J. (2013). Membrane potential and cancer progression. Front. Physiol. 4:185. 10.3389/fphys.2013.00185 23882223 PMC3713347

[B130] Yildirim S. Altun S. Gumushan H. Patel A. Djamgoz M. B. (2012). Voltage-gated sodium channel activity promotes prostate cancer metastasis *in vivo*. Cancer Lett. 323, 58–61. 10.1016/j.canlet.2012.03.036 22484465

[B131] Zhang W. Couldwell W. T. Simard M. F. Song H. Lin J. H. Nedergaard M. (1999). Direct gap junction communication between malignant glioma cells and astrocytes. Cancer Res. 59, 1994–2003. 10213512

[B132] Zhang W. DeMattia J. A. Song H. Couldwell W. T. (2003). Communication between malignant glioma cells and vascular endothelial cells through gap junctions. J. Neurosurg. 98, 846–853. 10.3171/jns.2003.98.4.0846 12691411

[B134] Zhao M. Chalmers L. Cao L. Vieira A. C. Mannis M. Reid B. (2012). Electrical signaling in control of ocular cell behaviors. Prog. Ret. Eye Res. 31, 65–88. 10.1016/j.preteyeres.2011.10.001 22020127 PMC3242826

[B135] Zoidl G. Dermietzel R. (2010). Gap junctions in inherited human disease. Pflugers Arch. 460, 451–466. 10.1007/s00424-010-0789-1 20140684

